# Extracellular vesicles isolated from hyperuricemia patients might aggravate airway inflammation of COPD via senescence-associated pathway

**DOI:** 10.1186/s12950-022-00315-w

**Published:** 2022-11-02

**Authors:** Xuanqi Liu, Zheng Li, Yang Zheng, Wenhao Wang, Peiqing He, Kangwei Guan, Tao Wu, Xiaojun Wang, Xuelin Zhang

**Affiliations:** 1 Shanghai Key Laboratory of Clinical Geriatric Medicine, Huadong Hospital Affiliated to Fudan University, Shanghai, 200040 China; 2 Department of Pulmonary and Critical Care Medicine, Zhongshan Hospital, Fudan University Shanghai Medical College, Shanghai, China; 3 Shanghai Institute of Infectious Disease and Biosecurity, Zhongshan Hospital, Fudan University, Shanghai, China; 4 Department of Thoracic Surgery, Huadong Hospital Affiliated to Fudan University, Shanghai, 200040 China; 5 Department of Traditional Chinese Medicine, Huadong Hospital Affiliated to Fudan University, Shanghai, 200040 China

## Abstract

**Backgrounds:**

Chronic obstructive pulmonary disease (COPD) is a major health issue resulting in significant mortality worldwide. Due to the high heterogeneity and unclear pathogenesis, the management and therapy of COPD are still challenging until now. Elevated serum uric acid(SUA) levels seem to be associated with the inflammatory level in patients with COPD. However, the underlying mechanism is not yet clearly established. In the current research, we aim to elucidate the effect of high SUA levels on airway inflammation among COPD patients.

**Methods:**

Through bioinformatic analysis, the common potential key genes were determined in both COPD and hyperuricemia (HUA) patients. A total of 68 COPD patients aged 50—75-year were included in the study, and their clinical parameters, including baseline characteristics, lung function test, as well as blood chemistry test were recorded. These parameters were then compared between the COPD patients with and without HUA. Hematoxylin & Eosin (HE), immunofluorescence (IF), and Masson trichrome staining were performed to demonstrate the pathological changes in the lung tissues. Furthermore, we isolated extracellular vesicles (EVs) from plasma, sputum, and bronchoalveolar lavage fluid (BALF) samples and detected the expression of inflammatory factor (Interleukin-6 (IL-6), IL-8 and COPD related proteases (antitrypsin and elastase) between two groups. Additionally, we treated the human bronchial epithelial (HBE) cells with cigarette smoke extract (CSE), and EVs were derived from the plasma in vitro experiments. The critical pathway involving the relationship between COPD and HUA was eventually validated based on the results of RNA sequencing (RNA-seq) and western blot (WB).

**Results:**

In the study, the COPD patients co-existing with HUA were found to have more loss of pulmonary function compared with those COPD patients without HUA. The lung tissue samples of patients who had co-existing COPD and HUA indicated greater inflammatory cell infiltration, more severe airway destruction and even fibrosis. Furthermore, the high SUA level could exacerbate the progress of airway inflammation in COPD through the transfer of EVs. In vitro experiments, we determined that EVs isolated from plasma, sputum, and BALF played pivotal roles in the CSE-induced inflammation of HBE. The EVs in HUA patients might exacerbate both systemic inflammation and airway inflammatory response via the senescence-related pathway.

**Conclusion:**

The pulmonary function and clinical indicators of COPD patients with HUA were worse than those without HUA, which may be caused by the increased airway inflammatory response through the EVs in the patient's peripheral blood. Moreover, it might mediate the EVs via senescence-related pathways in COPD patients with HUA.

**Supplementary Information:**

The online version contains supplementary material available at 10.1186/s12950-022-00315-w.

## Introduction

Chronic obstructive pulmonary disease (COPD) is one of the chronic airway diseases which is characterized by partially reversible airway limitation and lung tissue destruction. The prevalence of COPD is continuously rising, and it has become the third leading cause of mortality worldwide [1]. Considerable medical expenditure and research have been devoted to impeding the progression of the disease and episodes of acute exacerbation events, which has also added a huge burden to the public health system and deteriorated the life quality of COPD patients, especially the middle-aged and elderly patients [2]. It is widely accepted that cigarette smoke acts as an initial trigger for COPD. Also, it has been proposed that raised uric acid levels might be one of the poor prognostic factors in COPD patients [3]. Moreover, the level of serum uric acid (SUA) was found to be higher during the acute exacerbation stage of COPD [4]. Smilarly, other studies have also demonstrated the status of high SUA level might play a crucial role in the pathogenesis of COPD patients; however, the potential mechanism needs further in-depth investigation.

Worldwide, HUA is considered to be an independent risk factor for several diseases. The manifestation of HUA-induced renal damage consists of tubular injury, endothelial dysfunction, and oxidative stress [5]. Recent studies revealed that the high concentration of uric acid in peripheral circulation could induce intrarenal inflammation by activating the Nucleotide-binding oligomerization domain, leucine-rich repeat, and pyrin domain- containing 3 (NLRP3) inflammasome [6, 7]. Apart from renal diseases, the high SUA level was also found to be associated with an elevated risk of developing other chronic illnesses like cardiovascular diseases [8, 9] and respiratory diseases. Based on the current findings, HUA correlated strongly to pulmonary arterial hypertension (PAH), sleep breathing disorders (SBD), and COPD. The association between increased SUA level and COPD was observed in clinical epitemiology. HUA might lead to endothelial dysfunction and then induce pulmonary vasoconstriction and structure remodeling through chronic hypoxia and activation of the sympathetic nervous system [10]. At present, limited information is available regarding the potential targets and pathways during the high SUA-induced progression of COPD are still unclear.

Up till now, circulating EVs as promising biomarkers and potential therapeutics in COPD patients have been widely discussed [11, 12]. EVs, including those derived from inhaled bacteria, macrophages, epithelial cells, and endothelial cells, are related to COPD development and exacerbation to some extent [13]. Studies have found that both of the tissue resident cell (endothelial cells) and immune cells (monocytes) derived EVs was responsible for the inflammatory marker IL-6 in COPD [14]. Moreover,epithelial and endothelial cells derived EVs could regulate the immune balance of alveolar macrophages and exert influence on multiple lung diseases [15]. Meanwhile, endothelial cells were found to release more microparticles in response to various external irritants such as cigarette smoke, dust, and toxicity. Macrophages were also one of the crucial resources of EVs in the lungs. In detail, macrophages-derived EVs are related to antigen presentation, myeloid cell differentiation, and proliferation [13, 16]. However, few studies elucidated the accurate roles and biological process of EVs in the pathogenesis of COPD co-existing with HUA [13]. Based on the current literature, we hypothesize that the high SUA level might be associated with the pathology of lung tissue and chronic systemic inflammation in COPD patients, which might be mediated by EVs.

In the present study, the common underlying targets and pathways were observed among patients who co-existed with COPD and HUA according to the results of bioinformatics analysis. Extracellular signal transduction was assumed to participate in the pathological process of COPD. COPD patients enrolled in the study were divided into two groups based on the presence or absence of HUA, and chronic airway inflammation, airflow limitation, airway remodeling, and systemic inflammation were compared between these two groups. Furthermore, the inflammatory level in EVs derived from various body fluids (plasma, sputum, and bronchoalveolar lavage fluid (BALF)) derived from COPD cases was also verified. To explore the mechanism, we performed bulk RNA sequencing of HBE cells treated with EVs from subjects’ s plasma with or without HUA. Our study aimed to provide new insight into the relationship between high SUA levels with the severity of COPD, mediated by EVs.

## Methods

### Bioinformatics analysis

We used the Venn tool (https://bioinfogp.cnb.csic.es/tools/venny/) from the DisGeNET database (http://www.disgenet.org/) to identify the common genes in the two patient groups. Information related to functional enrichment, including Gene Ontology (GO) and Kyoto Encyclopedia of Genes and Genomes (KEGG) enrichment, was obtained from David online tool (https://david.ncifcrf.gov/) [17]. We developed a hub-key genes and protein–protein interaction (PPI) network through the software Cytoscape 3.8.0 and STRING (https://string-db.org/).

### Participant’s enrollment and human lung tissue samples collection

In the current study, a total of 68 patients aged 50–75 years were screened and eventually included in the analysis from January 1, 2021, to November 31, 2021, at Fudan University, affiliated with Huadong Hospital. COPD was diagnosed according to guidelines from the European Respiratory Society and the American Thoracic Society. The diagnostic criteria of COPD was FEV1 (forced expiratory volume in one second), less than 70% of the predicted value after bronchodilators. The diagnostic criteria of HUA were defined as SUA > 420 µmol/L. Patients with the following conditions should be excluded from the study: (1) different degrees of acute exacerbation of COPD in the past 3 months. (2) suffered from infectious airway disease in the past 3 months. (3) severe underlying disorders of kidney, liver, immune, cerebrovascular or hematopoietic system, cancer, or mental disorders. (4) participated in other clinical trials in the past 3 months. (5) serum creatinine level was > 1.5 mg/dL. (6) ALT level is more than twice the normal upper limit. (7) severe deformity or stiffness because of gouty arthropathy. (8) severe arrhythmia. (9) patients who consumed medications that contained aspirin (> 325 mg/d) or salicylate, azathioprine, 6-mercaptopurine, or hypouricemic medications. The study was approved by the Ethics Committee of Huadong Hospital, Affiliated with Fudan University (No. 20190037). All written informed consents were available, and this trial is registered with the Chinese Clinical Trial Registry (ChiCTR2000038257). The written informed consent was available according to the Declaration of Helsinki. The lung tissue samples were collected from patients undergoing pulmonary benign tumor approval from the Institutional Ethics Committee of Shanghai Huadong Hospital (2018K024).

### HBE Cells culture and CSE preparation

We acquired HBE cells from American Type Culture Collection (ATCC) and cultivated them in RPMI1640 medium with fetal bovine serum (all containing 5% FBS, Gibco, Invitrogen) at 37 °C in the presence of 5% CO2. CSE was obtained using the smoke of two cigarettes (Hong Shuang xi, a cigarette brand produced by Shanghai Tobacco Group, China). Tar (12 mg) and nicotine (1.0 mg) were collected in 15 mL of culture media using a 50 mL syringe. The resultant CSE solution was defined as 100% cigarette smoke extraction. It was frozen before preserving at -80 °C and filtered through a 0.22um filter to remove large particles before use. Subsequently, the CSE solution was diluted to the required concentration using culture media.

### Western blot

WB was performed on extracellular vesicle (EV) lysates obtained from plasma, BALF, and sputum of patients. Radioimmunoprecipitation assay (RIPA) buffer was used to prepare lysates of EVs. Thereafter, each sample was run in the NOVEX 10%–20% Tris–glycine gel (Invitrogen). In addition, we employed iBlot2 (Invitrogen) in transferring proteins onto polyvinylidene fluoride (PVDF) membranes (Millipore Sigma), followed by blocking with 5% bovine serum albumin (BSA, Sangon Biotech, Shanghai, China) within 0.1% Tween 20 (TBST) as blocking buffer for one hour.Then, we incubated membranes with diluted primary antibodies. The Image Lab software (Bio-Rad V5.2.1) was used for WB quantification, with Glyceraldehyde 3-phosphate dehydrogenase (GAPDH) being the reference. In the present work, the following primary antibodies (all were diluted at 1:1000) were adopted, including rabbit pAb Antitrypsin, rabbit pAb MUC1, rabbit pAb ELANE, rabbit pAb IL-6, rabbit pAb IL-8, rabbit pAb CD63, rabbit pAb TSG101, rabbit pAb Calnexin, rabbit pAb CDKN1A, rabbit pAb CDKN2A, rabbit pAb IL-1A, and rabbit pAb 53BP1. All antibodies were obtained from the brand ABclonal (Wuhan, China) (https://abclonal.com.cn/).

### The isolation of peripheral blood mononuclear cells (PBMCs)

PBMCs were obtained from the patients and separated with Ficoll density-gradient centrifugation. Later, we cultivated cells in RPMI-1640 medium supplemented with 10% FBS (Gibco).

### Reverse transcription-polymerase chain reaction (RT-PCR)

We isolated total cellular RNA by using a guanidinium thiocyanate reagent. Subsequently, cDNA was prepared from the isolated total RNA following specific instructions. We conducted RT-PCR with the green Polymerase Chain Reaction kit (std. Synergy Brands, Inc.) and amplified PCR products using ABI 7300. Then 2-ΔΔCt approach was utilized to determine gene level, with GAPDH being the reference. Primers used for RT-PCRs were: IL6, Fwd:5'-CCTTCTCCACAATACCCCCAGG-3'; Rev:5'-TGTGCCCAGTGGACAGGTTT-3'; IL8, Fwd: 5'-GTGCTGTGTTGAATTACGGA; Rev:5'-TTGACTGTGGAGTTTTGGC-3'; Antitrypsin, Fwd: 5'-ATGATGAAGCGTTTAGGCA-3'; Rev:5'-CAGGCAGGAAGAAGATGG-3'; ELANE, Fwd: 5'-CGACCCCGTAAACTTGCT-3'; Rev:5'-ACGTTGGCGTTGATGGT-3'; GAPDH, Fwd:5'-TGGGGTGATGCAGGTGCTAC-3'; Rev:5'-GGACACGGAAGGCCATACCA-3'.

### Enzyme-linked immunosorbent assay (ELISA)

ELISA was used to determine interleukin-6 (IL-6), IL-8, Antitrypsin and Elane levels in the plasma (X–Y Biotechnology, Shanghai, China). Plasma collected from patients was subjected to 3-min centrifugation at 500xg to remove debris. The kit contents were brought to the ambient temperature before starting the test. Sample diluent and standard were added to each blank well, followed by the addition of standards or samples of diverse doses (100 µL/well) in the rest wells. We used sealing tape to seal each reaction well, followed by a 90-min incubation at 36 °C in an incubator. We made the biotinylated antibody working solution 20 min before adding, which was diluted and added into each blank well, while the non-diluted solution was added into all rest wells (100 µL/well). The reaction well was sealed, followed by a 60-min incubation at 36 °C. Enzyme conjugate working solution was prepared 20 min before use at 22–25 °C in the dark, which was diluted and added into each blank well, whereas the non-diluted solution was added to all rest wells (100 µL/well) and incubated for 30-min at 36 °C in the dark. After heating, we used the microplate reader to measure absorbance. Then we added chromogenic substrate (TMB, 100 µL/well) into each well and incubated it for 15-min at 36 °C in the dark, followed by a stop solution (100 µL/well) into each well. After mixing, the OD450 value was recorded immediately (in 3 min), and target substance concentrations were measured in the samples.

### Cell viability assay on cells treated with different doses of CSE and EVs

After the isolation of CSE, the quality control of 100% CSE was performed by measuring the optical density at 320λ wavelength and a value of 1.158, and the intervention time and concentration was determined according to the previous research [18]. CBA assay was performed to evaluate the level of EVs from different subjects' plasma, and PBS was used to dilute. A Cell Counting Kit-8 (CCK-8, Beijing Solarbio Science and Technology Co., Ltd.) assay was conducted to assess cell viability after CES and EVs treatments at diverse doses. Cells (1 × 104 cells/ well) were cultivated within the 96-well plate for 24–96 h (Supplement 1). The microplate reader was used to measure the absorbance (OD) at 450 nm. All experiments were conducted three times. Therefore, we treated the HBE cells with CSE for 24 h and then EVs from subjects' plasma for another 24 h. The results of CCK8 have been show in the supplement.

### Immunohistochemistry (IHC), Masson staining, and Multiplex IF of lung tissues

We analyzed eight samples of lung tissue from COPD patients, including four samples of patients with normal SUA and four samples of patients with HUA. Lung tissue samples were collected from patients with benign lung tumors approved by the institutional ethics committee of Shanghai Huadong Hospital and voluntarily signed the informed consent. The specific inclusion criteria were consistent with those of the clinical cohort patients in this study. To analyze histology, we embedded lung tissues fixed with 4% paraformaldehyde (PFA) in paraffin and prepared the samples into 5-µm sections for HE and Masson staining. After thoroughly rinsing with normal saline, we fixed left lung tissue using 10% formalin solution neutral buffered. After xylene deparaffinization thrice (for 15, 5, and 10 min separately), we removed benzene with the 100%, 90%, 80%, and 70% ethanol gradient (10 min each). Each section was then rinsed for 5 min twice with distilled water, followed by 15-min hematoxylin (Recordbio, Shanghai, China) staining and washing with distilled water. Later, decolorization was done with 0.5% hydrochloric acid alcohol, rinsed for a 15-min period by distilled water, immersed in 70% and 80% ethanol in succession for a 10-min period, followed by 1-min eosin re-staining and 10-s 90% ethanol differentiation. Thereafter, we dehydrated sections for 10-min twice with 95% ethanol and then for 15-min twice with 100% ethanol. After clearing with xylene thrice (10, 15, and 15 min), we observed each section after neutral resin mounting. Before staining, we soaked each section for 30–60-s in distilled water, followed by the addition of hematoxylin nuclear staining solution to stain for 60-s, solution discarding and rinsing for 30-s. Sections were stained for a 30–60-s period by fuchsin acid staining solution, followed by solution discarding and rinsing for 30-s by the cleaning solution. Sections were then treated with the phosphomolybdic acid solution, followed by solution discarding following separation for 6–8 m. Sections were re-stained for a 5-min period with aniline blue re-staining solution; after that, the solution was discarded, and the sample was rinsed with anhydrous ethanol. We then sealed the dried sections for microscopic observation. The fibrosis area (%) in every group was measured through ImageJ. We took images under the inverted microscope (LSM 780) (Carl Zeiss, Jena, Germany) to identify the degree of chronic pulmonary inflammation, fibrosis, and emphysema. The three-color Fluorescence kit (Shanghai Recordbio Biological Technology, Shanghai, China) was utilized to co-stain elastase, antitrypsin, and 4’,6-diamidino-2-phenylindole (DAPI) through tyramide signal amplification (TSA) in line with the manufacturer’s instruction.

## IF staining of HBE cells

IF staining of CDKN1A and CDKN2A (Abclonal, China, shanghai) (recommended antibody dilution ratio range:1:50–1:200) was performed according to the description of antibody brand instructions (https://abclonal.com.cn/). HBE cells were intervened with CSE for 24 h and EVs for 24 h from the plasma of included patients with or without HUA. Cells were subjected to 4% PFA fixation and 1% donkey serum blocking. Later, primary antibodies against CDKN1A/CDKN2A (1:200) were used to incubate cells, followed by further incubation using Alexa-labeled anti-rabbit IgG (1:500). Later, cells were counter-stained with DAPI (Beyotime Biotech). We employed a fluorescence microscope to take photographs.

### HBE Cells RNA sequencing and analysis

Before collecting, we washed HBE cells using ice PBS twice. Then six samples from two groups (HBE cells intervened with EVs isolated from COPD patients with and without HUA) were collected. We isolated total RNA with TRIzol reagent as per instructions. Libraries were constructed, and mRNA was sequenced by Nuohe Zhiyuan Technology Company (Beijing, China) using Illumina NovaSeq 6000 high throughput sequencer. Moreover, we utilized DESeq (version 1.30.0) in identifying differentially expressed genes (DEGs). After adjusting the *p*-value with false discovery rate (FDR), we selected significant DEGs by *p* < 0.05 and ≥ twofold changes (FC) of thresholds. Fold change thresholds for all DEGs are placed in the Supplements. The sequencing data will be uploaded in SRA (The Sequence Read Archive).

### The isolation and characterization of EVs from plasma, sputum, and bronchoalveolar lavage fluid

The isolation of plasma and body fluid exosomes was done through RiboBio (Guangzhou, China) (https://www.ribobio.com/) as per the protocol. According to the protocol, the plasma sample was first transferred to a new tube and added 1/3 volume of RiboTM exosome isolation reagent (for plasma or serum), reversing the mixing or pipette mixing until the sample was completely mixed. Then, we kept it into a 4℃ refrigerator and let it stand for 30 min; Subsequently, at 4℃, 15,000 × G centrifugation for 2 min and then carefully suck the supernatant with a pipette; Finally, exosome obtained by centrifugation enters the subsequent experimental process.

According to the operator' manual [19], the BALF sample was removed from the low-temperature storage environment and place it on ice; At RT, 2000xG was centrifuged for 20 min to remove residual cells and debris; And we concentrated the BALF samples using the ultrafiltration tube (Millipore 10kd). Then we transferred the concentrated BALF to the new tube and added 1/3 volume of RiboTM exosome isolation reagent (for other body fluids). The sputum samples were homogenized using DTT and the supernatants were collected to the new tube, adding 1/3 volume of RiboTM exosome isolation reagent (for other body fluids).Then we reversed mixing or pipette mixing until the BALF sample, and sputum sample were completely mixed and put it in a 4 ℃ refrigerator and let it stand overnight; We subsequently transferred to 2 mL mixture to 2 mL centrifuge tube at 1500 × G centrifuge at 4 ℃ for 30 min and discarded the supernatant, getting a small part of exosome; Then we transferred 2 ml of mixed liquid to the same centrifuge tube at 1500 × G centrifuge at 4 ℃ for 30 min and discarded the supernatant. Finally, the EVs collected from plasma, sputum and BALF were resuspended with PBS for the subsequent experimental process.Later, bicinchoninic acid (BCA) assay was conducted to determine protein content in EVs from plasma, sputum, and BALF. The morphology of EVs was observed under a transmission electron microscope (TEM) at 80 kV acceleration voltage. The specific surface protein markers CD63 and TSG101 of EVs were determined using WB.

### Mitochondrial DNA (mtDNA) analysis

mtDNA content in PBMCs from COPD patients with or without HUA was determined by RT-PCR. Total DNA was isolated using a DNA assay kit (Tiangen, Beijing). Primer Mix from human mitochondrial DNA probe fluorescent quantitative PCR kit (X–Y Biotechnology, Shanghai, China, shxysw.bioon.com.cn). RT-PCR was performed on the StepOne real-time PCR system using Platinum SYBR Green qPCR Supermix-UDG. Reactions were prepared as per specific protocols within the 20-µL system (X–Y Biotechnology).

### Statistical analysis

Results were displayed in the form of mean ± SD. Independent variables for each group or condition are presented in figure legends. Different statistical methods, including student’s t-test, Mann-whiney-test, and ANOVA, were adopted as indicated.

In our study, a two-sided *P* < 0.05 was considered to be statistical significance. *P* < 0.05(*), *P* < 0.01(**), and *P* < 0.001(***) imply significance. Statistical analysis was done with STATA 15.0 and Prism 8.0.

## Results

### The potential targets and pathways of interaction between COPD and HUA

**Fig. 1:**
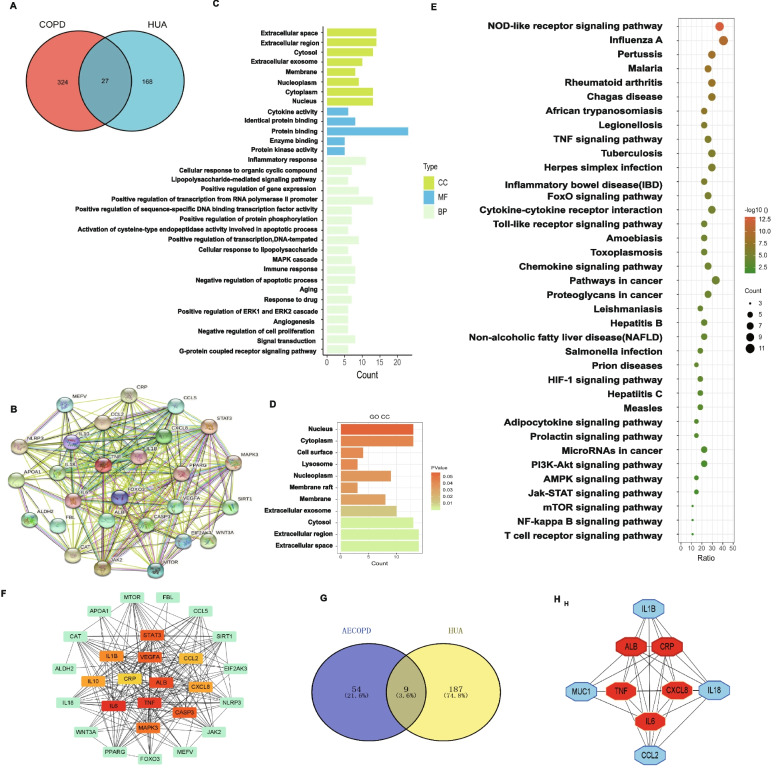
The potential targets and pathways between COPD and HUA. **A** The Venn diagram showing the shared genes between COPD and HUA. **B** The protein interaction network of 27 common genes. **C** The bar plot showing the GO enrichment of core common genes including CC, MP and CM. **D** The bar rplot showing the cellular component terms in GO enrichment. **E** The bubble plot showing the KEGG enrichment of common genes. **F** The hub-genes among the core 27 shared genes. **G** The Venn diagram showing the shared genes between AECOPD and HUA. **H** The hub-genes among the common 9 genes. *Abbreviation*: CC, cell component; BP, biological process; MF, molecular function; AECOPD, Acute Exacerbation of Chronic Obstructive Pulmonary Disease; HUA, hyperuricemia; COPD, chronic obstruction pulmonary disease

Based on the bioinformatics analysis, we identified 27 common genes between HUA and COPD from the disease database (DisGeNET) (Fig. 1A). We also established the PPI network for displaying the relationship among these overlapped 27 genes (Fig. 1B). Thereafter, functional enrichment analysis was performed using these key genes, and the top key GO terms and detailed important cellular component terms were respectively summarized in Fig. 1C and D. Next, we conducted a KEGG enrichment analysis, and the results revealed 37 significantly enriched pathways in both HUA and COPD. The leading KEGG pathways were identified as “NOD-like receptor signal pathway”, "TNF signaling pathway", "FoxO signaling pathway," and "Toll-like receptor signaling pathway", which were most closely related to the immune system and inflammation (Fig. 1E). We further conducted the hub gene network profile analysis to demonstrate the interaction between critical and subcritical genes and corresponding effects (Fig. 1F). Moreover, we explored the association between acute exacerbation of chronic obstructive pulmonary disease (AECOPD) and HUA (Fig. 1G). A total of 9 key inflammation response-related genes were determined, especially IL-6 and CXCL8, for further analysis (Fig. 1H).

### Clinical indicators in included COPD patients

**Fig. 2:**
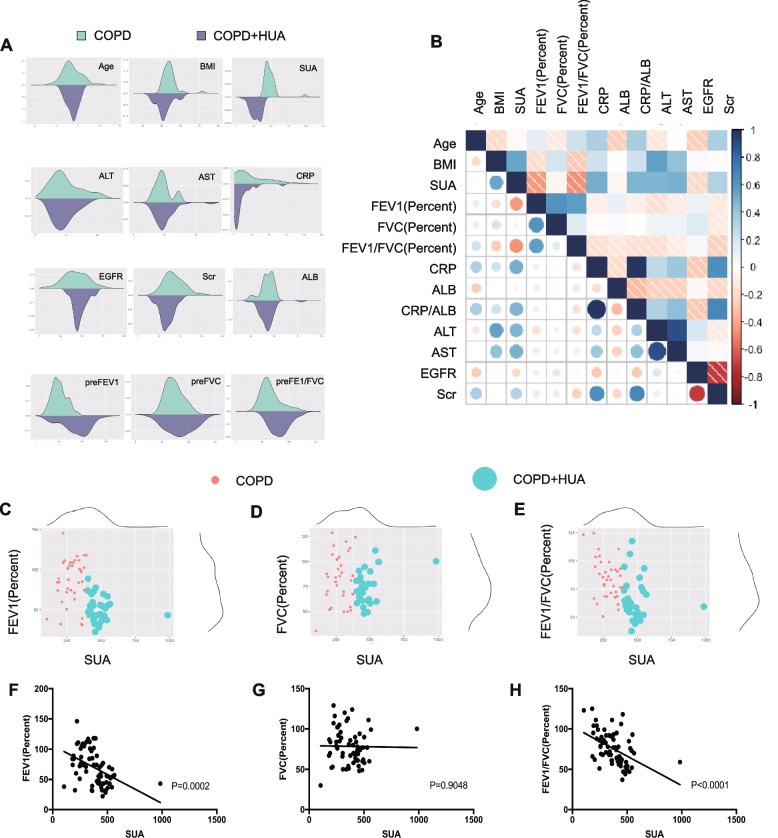
Clinical features of the included COPD patients. **A **Density distribution showing the difference of clinical indicators between COPD patients with or without HUA groups. **B** Correlation coefficient matrix indicating the correlation between clinical parameters including baseline features, blood biochemistry and lung function test among COPD patients. **C**-**H **Scatter diagram and linear regression showing the level of serum uric acid and lung function test FEV1percent (**C**, **F**), FVC percent (**D**, **G**) and FEV1-FVCpercent (**E**, **H**). *Abbreviation*: COPD, chronic obstruction pulmonary disease; HUA, hyperuricemia; SUA, serum uric acid; BMI, body mass index; FEV1, forced expiratory volume in one second; FVC, forced vital capacity; CRP, C-reactive protein; ALT, alanine transaminase; AST, aspartate transaminase; ALB, albumin; EGFR, estimated Glomerular Filtration Rate; SCR, serum creatinine

The included COPD patients were divided into two groups according to whether they were complicated with HUA. Clinical characteristics of COPD cases with and without HUA are presented in Table 1 and Fig. 2A. There was no significant difference in sex and age between the participants in these two groups. Compared with COPD patients without HUA group, we found that COPD patients with HUA had significantly higher BMI (*P* = 0.008). The results also revealed that severe metabolic disorders tend to occur in COPD patients with co-existing HUA. To investigate the role of HUA in the lung function change of COPD cases, we analyzed the parameters of pre FEV1% (*P* = 0.0002), preFVC %, and preFEV1/FVC% (*P* < 0.0001) between these two groups, and COPD patients with HUA showed worse airway dysfunction and airflow limitation.

**Table 1:** Clinical features and baseline characteristics between two groups

	**Total**	**COPD(*N* = 37)**	**COPD + HUA(*N* = 31)**	***P*-value**
Age	68.41 ± 8.77	68.65 ± 1.04	68.13 ± 2.00	0.8099
Gender (Male/Female)	55/13	27/10	28/3	0.070
BMI	22.81 ± 4.38	21.55 ± 0.71	24.33 ± 0.73	0.0080
**Pulmonary function**				
PreFEV1	69.86 ± 28.97	85.61 ± 4.64	51.06 ± 2.82	< 0.0001
PreFVC	78.51 ± 21.78	83.15 ± 4.34	73.04 ± 3.06	0.0703
PreFEV1/FVC	75.42 ± 20.28	83.86 ± 2.94	65.33 ± 3.32	0.0001
Post-FEV1	69.99 ± 19.21	82.68 ± 14.56	60.97 ± 15.357	0.4399
PostFVC	78.45 ± 25.87	83.83 ± 17.64	74.10 ± 10.71	0.6077
Post-FEV1/FVC	75.56 ± 23.44	84.43 ± 17.16	66.74 ± 14.40	0.7026
**Hemogram indices**				
SUA	376.23 ± 133.04	284.46 ± 11.57	485.75 ± 18.67	< 0.0001
ALT	20.86 ± 17.79	18.58 ± 1.14	23.58 ± 4.53	0.2519
AST	21.53 ± 16.96	20.02 ± 1.14	23.32 ± 4.32	0.4294
EGFR	92.92 ± 26.68	99.00 ± 3.93	85.86 ± 5.15	0.0436
Scr	79.08 ± 36.11	67.62 ± 2.62	92.75 ± 8.53	0.0035
CRP	22.06 ± 42.72	9.39 ± 2.15	37.19 ± 10.53	0.0066
ALB	42.28 ± 7.77	42.27 ± 1.40	42.30 ± 1.23	0.9898
CRP/ALB	0.62 ± 1.62	0.21 ± 0.04	1.11 ± 0.42	0.0218

*Abbreviation:* *COPD* chronic obstructive pulmonary disease, *HUA* hyperuricemia, *SUA* serum uric acid, *BMI* body mass index, *FEV1* forced expiratory volume in one second, *FVC* forced vital capacity, *CRP* C-reactive protein, *ALT* alanine transaminase, *AST* aspartate transaminase, *ALB* albumin, *EGFR* estimated Glomerular Filtration Rate, *SCR* serum creatinine

There's no significant difference of post FEV1%, post FVC %, and post FEV1/FVC% (Table 1) in our study. It may be absribed to the strong heterogenuity and high standard deviation. The enlarged size of participants should be enrolled in the future. Moreover, blood biochemistry test results revealed a higher value of inflammatory markers like C-reactive protein (CRP) (*P* = 0.0066) in COPD patients with HUA (Table 1, Fig. 2A) than those without HUA. We also found that the ratio of CRP/(albumin) ALB was elevated in COPD patients with HUA compared with COPD patients without HUA (*P* = 0.0218), which is in agreement with our bioinformatics analysis (Fig. 1F). The correlation analysis of clinical variables is depicted in Fig. 2B. The scale bar represents the correlation coefficient between clinical variables. Blue indicates positive correlation, red indicates negative correlation; The color depth indicates the value of the coefficient. The darker the color, the stronger the correlation. As shown in Fig. 2B, the level of SUA was negatively associated with lung function in COPD patients and positively associated with CRP, CRP/ALB, alanine transaminase (ALT), and aspartate transaminase (AST), which suggested that high SUA levels might play a negative role in the process of COPD. Furthermore, the scatter diagram displays the association of SUA with pulmonary function (Fig. 2C-H). Hence, it was observed that in COPD patients with HUA, respiratory health-related clinical indicators, as well as systematic inflammatory indicators, were deranged compared with COPD patients without HUA.

### The inflammatory status of lung tissue and peripheral circulation in COPD patients with or without HUA

**Fig. 3:**
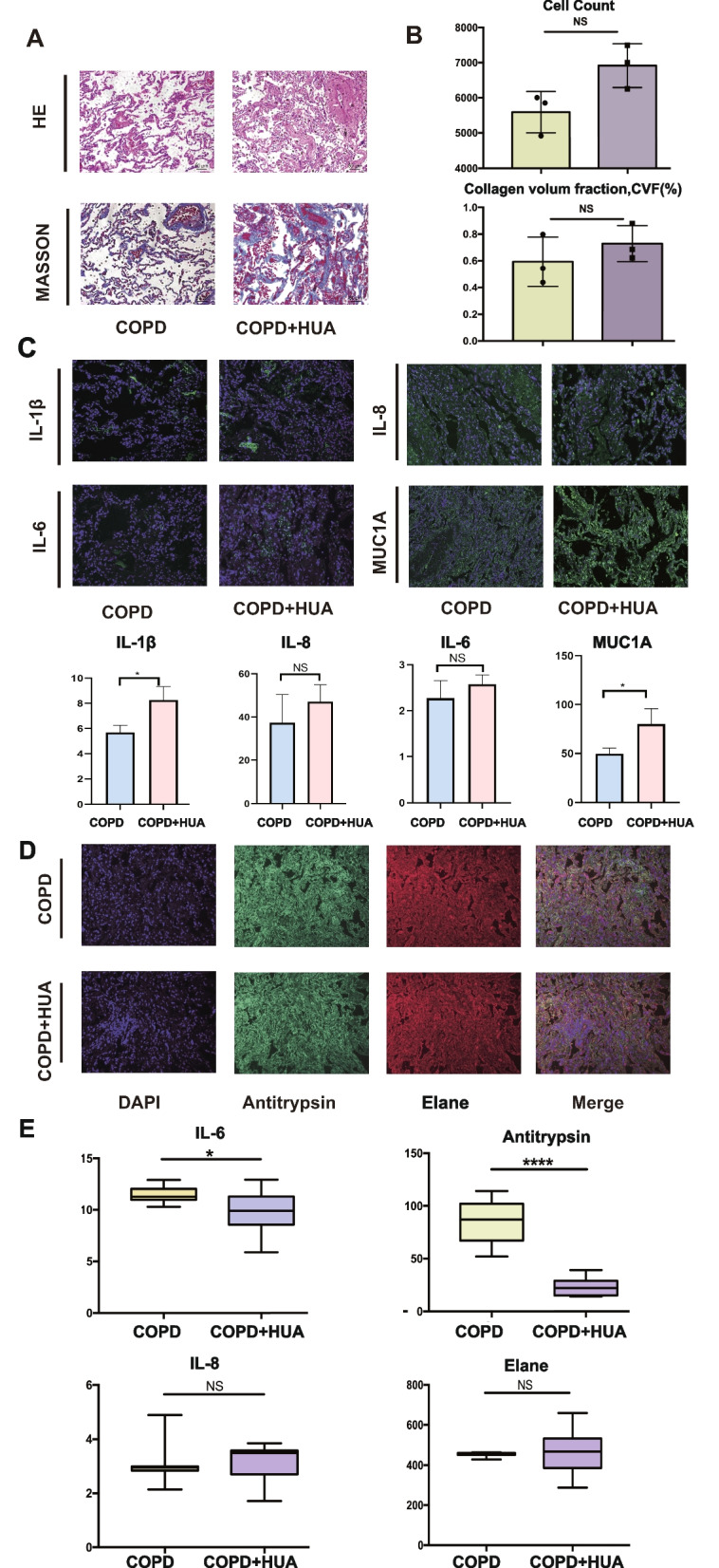
The comparison of inflammation status in lung tissue and peripheral blood between COPD patients with or without HUA. **A** HE staining detected alveolar cells morphology (X20) and MASSON staining of lung tissue (X20). Scale bars, 50 μm. **B** Quantification of cell counts according to HE staining and fibrotic area of difference groups by MASSON staining (X20). Scale bars, 50 μm. **C** Expression of inflammatory factors, IL-1β, IL-6, IL-8 and MUC1 were detected in lung by multiplex immunofluorescences(X20). Scale bars, 50 μm. The immunofluorescence quantification of mean fluorescence intensity was indicated in the bar plot. **D **Multiplex immunofluorescences of location of Antitrypsin and Elane in lung tissues(X20). Scale bars, 50 μm. **E **The level of IL-6, IL-8, Antitrypsin and Elane in plasma from two groups detected by ELISA (unpaired t test, *P < 0.05 and ****P < 0.001). *Abbreviation*: COPD, chronic obstruction pulmonary disease; HUA, hyperuricemia; HE, Hematoxylin & Eosin; IL-6, interleukin-6; IL-8, interleukin-8; IL-1β, interleukin-1 beta; DAPI, 4’,6-Diamidino-2’-phenylindol; MUC1, mucin 1

In order to compare the pathological changes between COPD patients with or without HUA, we collected the lung tissue samples and performed HE staining and MASSON staining, respectively. Compared with COPD patients without HUA, HE staining (Fig. 3A) demonstrated bronchial wall thickening as well as increased infiltration of inflammatory cells within submucosa in the group of COPD patients with HUA. According to the quantitative results, we found that MASSON staining, including cell count and collagen volume fraction, did not reveal any significant difference between the two target populations (Fig. 3B). As shown in Fig. 3C, the co-localization and co-expression of canonical airway inflammatory factors, including IL-1β, IL-6, IL-8, and MUC1, were analyzed by IF cytochemistry. The quantitative analysis based on IF assay showed that the intensity of IL-1B and MUC1 was increased, whereas the intensity of IL-6 and IL-8 was changed without significance (Fig. 3C). Furthermore, Multiplex IF analysis was also performed to measure the balance of the protease system. The diminished intensity of antitrypsin in COPD patients with HUA was observed (Fig. 3D) when compared with those without COPD. Apart from the inflammatory factors in lung tissue, the expression level of IL-6, IL-8, Elane, and antitrypsin in plasma was detected by ELISA (Fig. 3E). According to the results, differences in Elane and IL-8 levels of both groups were not significant. COPD-HUA group had a significantly upregulated level of IL-6(*P* < 0.05) while a remarkably downregulated level of antitrypsin(*P* < 0.0001).

### The different levels of inflammatory factors in extracellular vesicles of COPD patients with and without HUA

**Fig. 4:**
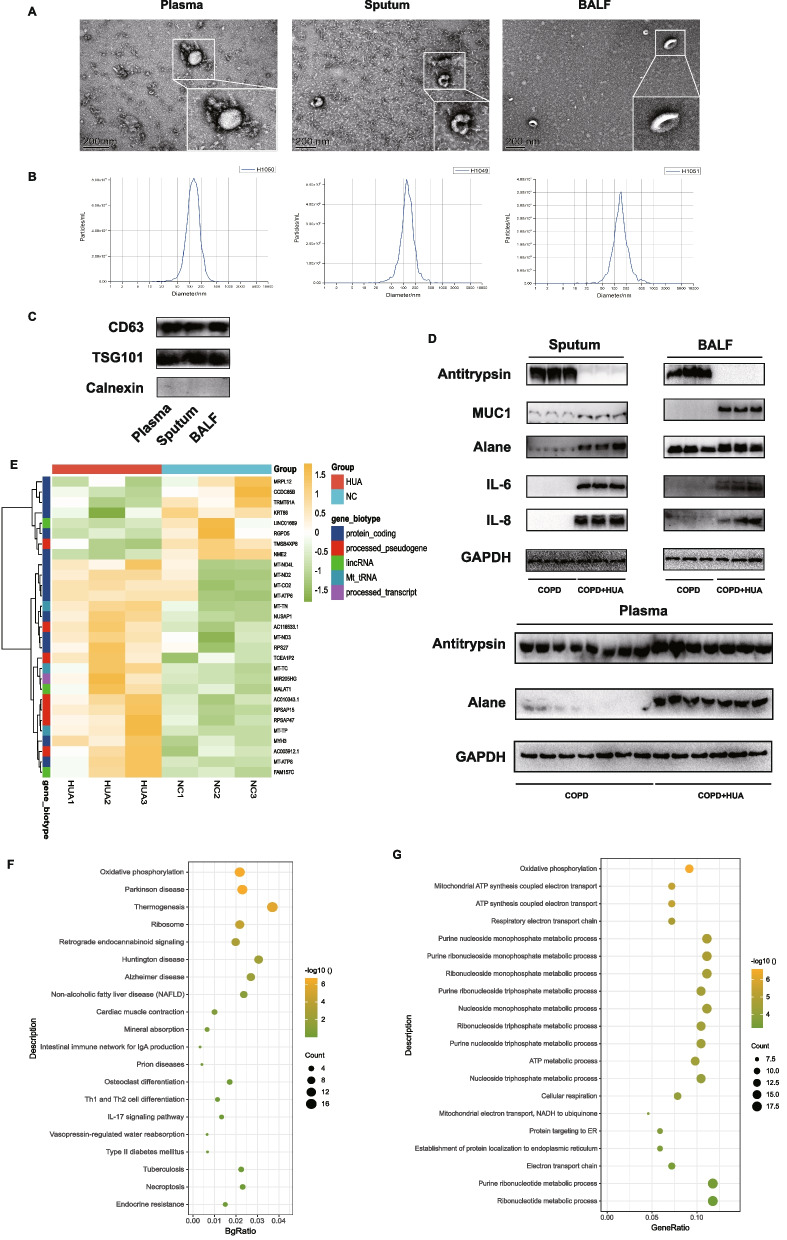
Identification of plasma, sputum and bronchoalveolar lavage fluid (BALF) derived EVs from included COPD patients. **A** Morphology of extracellular vesicles by electron microscope and transmission electron microscope. **B **Exosome nanoparticle tracking analysis. The mean size of EVs in sputum is 124.4 nm; EVs in BALF is 142.1 nm; EVs in serum is 124.1 nm. **C **Identification of characteristic marker including TSG101 and CD63 by western blot. **D** Level of IL-6, IL-8, Elane, Antitrypsin and MUC1 in extracellular vesicles derived from sputum, BALF and plasma of COPD patients examined by western blot. **E **Heatmap indicating the results of mRNA expression of significant difference genes between two groups (HBE treated CSE and subsequent EVs isolated from patients with or without HUA). **F**-**G** KEGG enrichment (**F**) and GO enrichment (**G**) analysis of these significant differentially expressed genes. *Abbreviation*: BALF, bronchoalveolar lavage fluid; TSG101, tumor susceptibility gene101; CD63, lysosomal membrane-associated glycoprotein 3; MUC1, mucin 1; EVs, extracellular vesicles; COPD, chronic obstruction pulmonary disease

To further explore the role of EVs in the pathological process of COPD patients co-existed with HUA, we first compared the level of multiple inflammatory factors and proteases in EVs. The extracellular vesicles isolated from plasma, BALF, and sputum in COPD patients were respectively identified by transmission electron microscopy, exosome nanoparticle tracking analysis (NTA), and WB (Fig. 4A-C). Surface markers of EVs were verified using the marker of Calnexin, CD63 and TSG101 (Fig. 4C). The expression of inflammatory factors (IL-6 and IL-8), MUC1, Elane, and antitrypsin were compared between these two groups. COPD cases with HUA experienced significantly elevated IL-6 and IL-8 within sputum EVs (Fig. 4D) when compared with COPD cases without HUA. A similar trend was also observed in EVs from BALF that higher expression of Elane and MUC1 was found in COPD patients with HUA compared with those without. However, the level of antitrypsin was lower among COPD patients with HUA (Fig. 4D). Further, in the part of vitro experiments, we treated the HBE cells with double stimulus, including CSE for 24 h and subsequent EVs from patients' plasma for another 24 h. Finally, we harvested the HBE cells to perform bulk RNA sequencing (Fig. 4E). A heatmap was constructed to display the different mRNA expression patterns between these two groups (HBE cells treated with HUA patients' EVs and normal controls' EVs in plasma). Consequently, all differentially expressed genes were selected for further analysis. These genes we discovered mainly belong to the mitochondria family of genes. Through GO and KEGG enrichment analysis, it implicated that oxidative phosphorylation might be involved in the high uric acid-induced pathological process in HBE cells (Fig. 4F-G).

### The pathology of COPD may be associated with the mtDNA in EVs via a senescence-related pathway

**Fig. 5:**
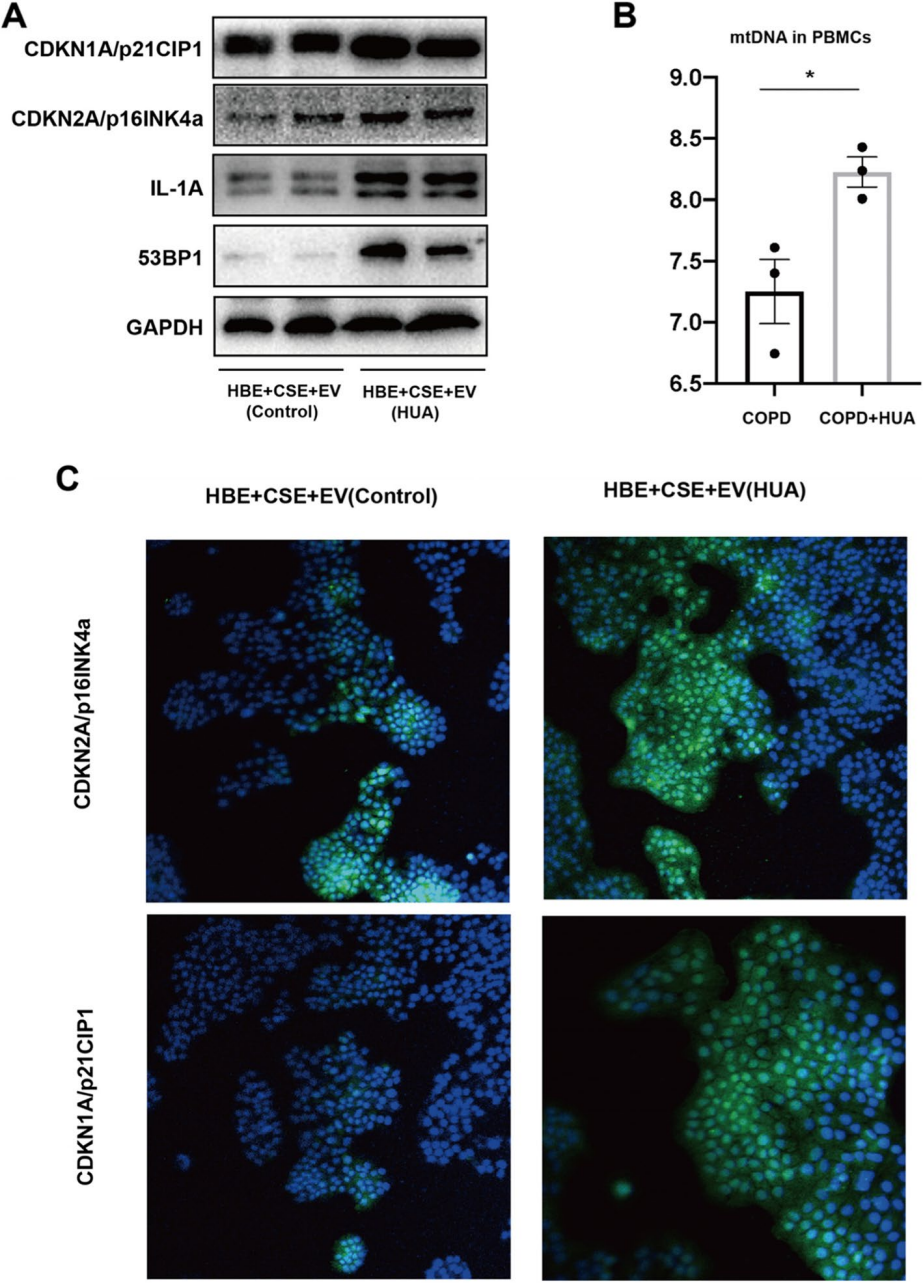
The pathology of COPD may be associated with mtDNA in EVs via a senescence related pathway. **A** Identification of senescence related protein including CDKN1A, CDKN2A, IL1A and 53BP1 in HBE treated with CSE and EVs from healthy controls and HUA patients was performed by western blot. **B** RT-PCR analysis of mtDNA content in PBMCs derived from COPD patients with or without HUA was compared using RT-PCR (unpaired t test, *P < 0.05). **C** IF microscopy analysis of CDKN1A and CDKN2A expression in HBE cells treated with CSE and subsequent EVs from patients with or without HUA(X20). Scale bars, 500 μm. *Abbreviation*: PBMC, peripheral blood mononuclear cell; CDKNI1A, cyclin-dependent kinase inhibitor 1A; CDKN2A, cyclin-dependent kinase inhibitor 2A; 53BP1, recombinant Tumor Protein p53 Binding Protein 1; IL-1A, Human IL-1 alpha protein; HBE, human bronchial epithelial; CSE, cigarette smoke extract; EVs, extracellular vesicles

To determine whether the EVs from HUA patients aggravate the process of CSE included inflammation response in HBE cells through senescence-related pathways, we explored several canonical markers of senescence, including cyclin-dependent kinase inhibitor 1A(CDKNI1A), cyclin-dependent kinase inhibitor 2A(CDKN2A), 53BP1, and IL1A in vitro experiments. The expression of CDKN1A, IL1A, and 53BP1 was significantly elevated in the plasma EVs of HUA patients compared with controls (Fig. 5A). The content of mtDNA, denoted in the form of the copy number of mtDNA relative to nuclear DNA, is one of the important indicators of mitochondrial function. As shown in Fig. 5B, the mtDNA amount was significantly higher in PBMCs from COPD cases who co-existed with HUA compared with those without (*P* < 0.05). Additionally, we conducted an IF assay to indicate the co-location of CDKN2A and CDKN1A in HBE after the co-stimulation of CSE and EVs. It indicated that the level of the two genes was higher in the HUA group when compared with controls (Fig. 5C). The results implicated that EVs from the HUA patients' peripheral circulation might accelerate the pathological process of COPD, including airway inflammation and tissue destruction through the senescence concerned pathways.

## Discussion

COPD has become a major health issue worldwide, leading to morbidity and mortality. It is a typical example of lung disease caused by the imbalance of protease and anti-protease [20, 21]. COPD has the features of airway obstruction, chronic airway inflammation, and emphysema, which can cause cough, respiratory failure, and shortness of breath. Inflammatory response results in repetitive tracheal wall injury and repair, resulting in tracheal structural remodeling, scar formation, and increasing collagen levels [22]. Such pathological alterations lay the major pathological basis for airflow obstruction in COPD [23]. Though UA is a major antioxidant, still in certain conditions, it acts as an inflammatory index as well as a danger signal of lung function loss [24, 25]. High SUA level is associated with COPD disease progression, but underlying mechanism and common pathway have not been determined [26]. SUA can be produced by injured cells for forming urate crystals, and the latter can be utilized by the immune system for producing interleukin (IL)-1. Danger signals and IL-1 play an important role in the course of COPD [27]. In this study, bioinformatics analysis was used to derive IL-6, IL-8, IL-1 β and MUC1 may play a potential role in the exacerbation of COPD disease progression by high uric acid levels, which is also consistent with the results of current studies [28–30].

Exosomes are usually made of four transmembrane proteins, transmembrane receptors, membrane transporters, specific proteins, signal transduction proteins, and nucleic acids (miRNA, mRNA, DNA) [31]. Once entering the extracellular space, exosomes are absorbed by adjacent cells or enter the blood circulation to reach distant targets and release genetic materials, protein, and lipid mediators. In recent years, it has been observed that exosomes can transmit information between cells and regulate the function of receptor cells through paracrine and other pathways [19, 32]. Physiologically, exosomes have been proved to be the key mediators in maintaining the steady-state function of complex thin-walled lung tissue and airway structure [33]. In addition to being a biomarker for diagnosis and prognosis, exosomes have also been reported to play a role in the chronic inflammatory response of the airway and lung tissue and induce the migration of inflammatory cells [19], indicating their effect on COPD genesis and progression [22, 34].

Aging is associated with a gradual loss of pulmonary function because of aggravated cell aging, reduced regeneration ability, and damaged natural host defense [35]. Under physiological conditions, aging can result in lung elasticity loss and enlarge the alveolar spaces among the old population, a phenomenon called 'senile emphysema', and during COPD, peripheral airway fibrosis and alveolar wall destruction are observed [36, 37]. Nonetheless, COPD shows distinct age-related characteristics, such as increased cell aging, exhausted stem cells, aggravated oxidative stress, and immune senescence. Cell aging results in the decline of alveolar elasticity, the increase of physiological and functional residual capacity, and the overinflation of alveoli. The overinflation of alveoli aggravates the decline of pulmonary function [38]. It has been reported that unrepaired DNA damage will accelerate the rate of aging [39]. mtDNA begins to damage over time, and the ability of cells to produce energy is gradually lost, leading to aging [40]. Mitochondria, known as the power plant of cells, produce 90% of chemical energy to maintain cell survival [41]. In humans, mitochondrial impairment may result in age-associated disorders. mtDNA depletion is also related to diabetes, mitochondrial disorders, cancers, or age-associated nervous system disease [42–44]. In our study, we included patients with senile emphysema, among whom the main clinical manifestations were chest tightness and persistent wheezing, to determine whether elderly patients with HUA may accelerate the disease progression of COPD through senescence-related pathways.

It has been recognized that the comorbidity of COPD exerts a great influence on the mortality of COPD patients. Of these, metabolic disorders are often overlooked. The previous studies exploring the relationship between COPD and HUA were indirect, and the corresponding results were ambiguous. In a study, it was found that the level of SIRT1 in PBMCs of COPD patients was higher [45]. High uric acids concentration in peripheral blood has been proved to be associated with severe exacerbation events and lung function among no matter in adolescent or adult asthma patients [46]. Additionally, the dysregulation of mitochondria morphology and function in airway epithelial cells have been proposed. Therefore, we aim to bridge the two diseases via the transfer of EVs and eventually target the pathological change of mtDNA.

Senescence associated secretory phenotype (SASP) refers to the hypersecretion of senescent cells, which can secrete many bioactive molecules, including cytokines, chemokines, growth factors, and proteases, and participate in a variety of physiological and pathological processes. These secretory factors are collectively referred to as SASP factors [47, 48]. We observed that the typical SASP secretory factors IL-6/-8/-10 were markedly increased in the peripheral blood of patients with HUA compared to control. As an important medium of intercellular communication, the genetic material in exons mediates intercellular communication through direct action or the formation of RNA protein complexes [49]. Meanwhile, in the present study, the intervention of EVs derived from patients with HUA could increase the expression of cell senescence specific markers (CDKN1A/p21, CDKN2A/p16 and IL-1A) [50, 51] and DNA damage response marker 53BP1 proteins [52] in COPD cell model. Studies have confirmed that mtDNA in outer vesicles can activate the alert state of receptor cells [53, 54]. DNA transfer in exosomes has a functional effect on recipient cells and can regulate the unique gene expression pathway in normal recipient cells [55]. At first, in vitro RNA sequencing results indicated that genes associated with mitochondrial dysfunction were altered in bronchial epithelial cells after exosome intervention. In addition, we found more mtDNA in the plasma exosomes of COPD patients with HUA.

In this study, we conclude that there is a correlation between high SUA level and airway inflammation in COPD patients through bioinformatics analysis and clinical data results, which may be related to EVs in the patient's external circulation. However, there are still several limitations in the current study. First, most of the participants in our cohort were male; comparative lack of female patients can create bias when applying our conclusion to a larger population to some degree. What’s more, as the high incidence of COPD is the middle-aged and elderly people over 40 years old, the population in our study is lack of contrast of the young group. In future studies, we will supplement the young COPD patients with or without HUA in order to more clearly define the potential effect of high SUA levels in the progress of COPD. In addition, we will also expand the sample size and conduct a prospective cohort study to observe the impact of dynamic change in SUA level on airway inflammation degree and lung function in COPD patients.

In sum, the pulmonary function of COPD patients with HUA was worse than that of patients without HUA, which may be caused by the increased inflammatory response of pulmonary airway epithelial cells by the EVs in the patient's external circulation. This research suggests that an increased SUA level can be an unfavorable factor in cytokine upgrading and organ damage associated with senile emphysema and that mtDNA encapsulated in plasma exosomes may participate in the process of bronchial epithelial cell damage and play a biological role. The specific mechanism and how exosomes transfer mtDNA need to be explored in future research.

## Supplementary Information

**Additional file 1.** 

**Additional file 2.**

## Data Availability

The raw data supporting the conclusion in the article will be made available by the authors without reservation.

## Abbreviations

COPD: Chronic obstructive pulmonary disease
HUA: Hyperuricemia
SUA: Serum uric acid
HE: Hematoxylin & Eosin
IF: Immunofluorescence
DAPI: 4’,6-Diamidino-2’-phenylindol
BALF: Bronchoalveolar lavage fluid
HBE: Human bronchial epithelial
CSE: Cigarette smoke extract
EVs: Extracellular vesicles
NTA: Nanoparticle tracking analysis
RNA-seq: RNA-sequencing
WB: Western blot
NLRP3: NOD-like receptor thermal protein domain associated protein 3
PAH: Pulmonary arterial hypertension
SBD: Sleep breathing disorders
GO: Gene ontology
KEGG: Kyoto Encyclopedia of Genes
PPI: Protein-protein interaction
BMI: Body mass index
FEV1: Forced expiratory volume in one second
FVC: Forced vital capacity
ATCC: American Type Culture Collection
FBS: Fetal bovine serum
RIPA: Radioimmunoprecipitation assay
PVDF: Polyvinylidene fluoride
GAPDH: Glyceraldehyde 3-phosphate dehydrogenase
RT-PCR: Reverse Transcription-Polymerase Chain Reaction
ELISA: Enzyme-linked immunosorbent assay
TMB: Chromogenic substrate
IHC: Immunohistochemistry
PFA: Paraformaldehyde
FDR: False discovery rate
FC: Fold change
BCA: Bicinchoninic acid
TEM: Transmission electron microscope
AECOPD: Acute exacerbation of chronic obstructive pulmonary disease
CRP: C-reactive protein
ALT: Alanine transaminase
AST: Aspartate transaminase
ALB: Albumin
EGFR: Estimated glomerular filtration rate
SCR: Serum creatinine
IL-6: Interleukin-6
IL-8: Interleukin-8
IL-1β: Interleukin-1 beta
TSG101: Tumor susceptibility gene101
CD63: Lysosomal membrane-associated glycoprotein 3
MUC1: Mucin 1
CDKNI1A: Cyclin-dependent kinase inhibitor 1A
CDKN2A: Cyclin-dependent kinase inhibitor 2A
53BP1: Recombinant Tumor Protein p53 Binding Protein 1
IL-1A: Human IL-1 alpha protein
SASP: Senescence-associated secretory phenotype
