# Supplementary material for: Extracellular vesicles isolated from hyperuricemia patients might aggravate airway inflammation of COPD via senescence-associated pathway
Source: J Inflamm (Lond). 2022 Nov 2;19:18. doi: 10.1186/s12950-022-00315-w (PMC9628085; doi:10.1186/s12950-022-00315-w)
Supplement: Supplementary file 1 — Additional file 1. [file 12950_2022_315_MOESM1_ESM.docx]

**Supplement 1. CCK8 assay of the HBE availability.**

**
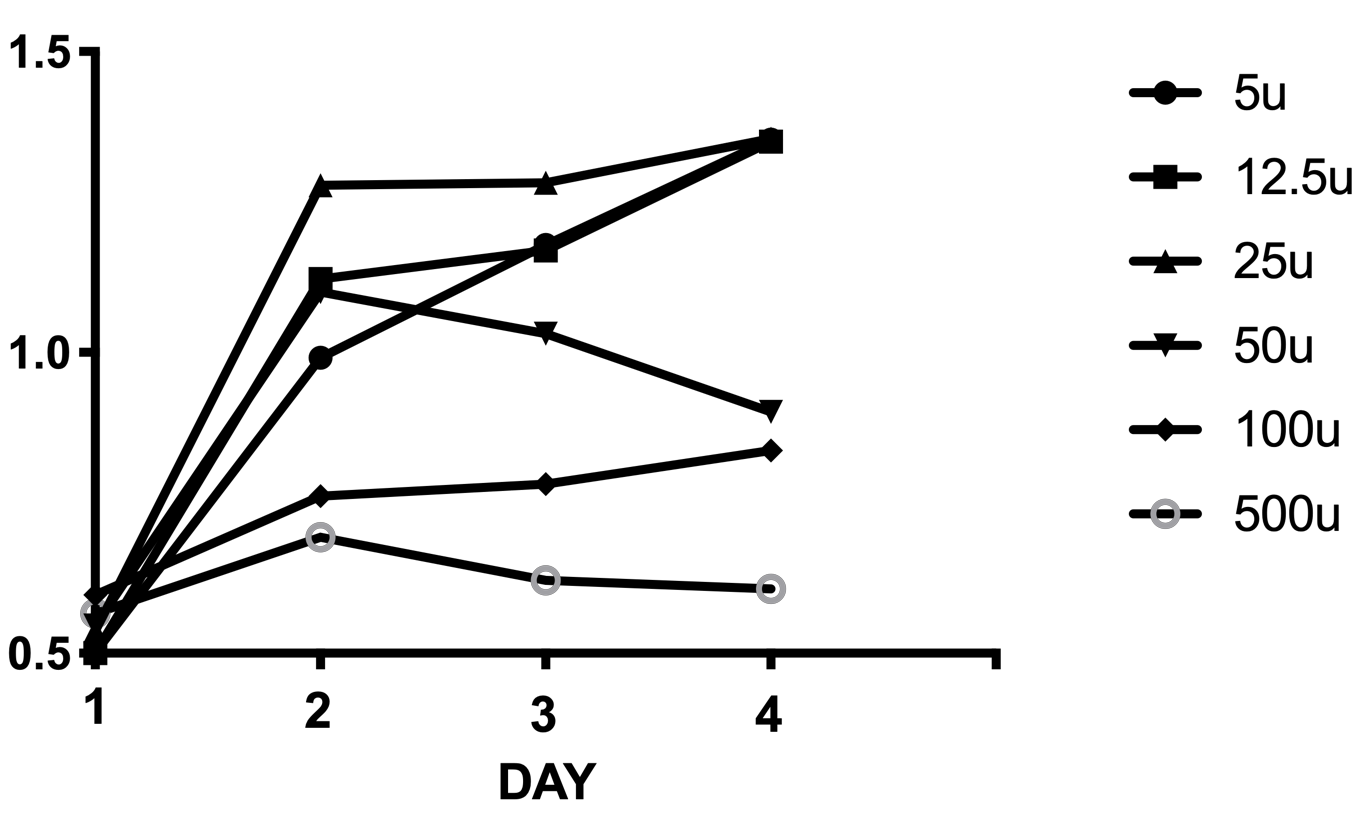
**

HBE treated with 10%CSE for 24h and then treated with diverse concentration of extracellular vesicles harvested from plasma for 24h-96h were evaluated by CCK8. ***Abbreviation***: CCK8, Cell Counting Kit-8; HBE, human bronchial epithelial; CSE, cigarette smoke extract.

**Supplemental 2.** The full list of the differentially expressed genes between HBE stimulated with CSE and extra vesicles from normal control group or HUA group via bulk RNAseq.

| Gene_name | HUA | NC | log2FoldChange | P-value | Adjusted P-value |
| --- | --- | --- | --- | --- | --- |
| MT-ATP8 | 462.0066074 | 228.8732435 | 1.014208473 | 1.86E-08 | 0.000537861 |
| AC010343.1 | 298.3662628 | 139.2550064 | 1.100223605 | 7.65E-08 | 0.001109114 |
| FAM157C | 220.5801951 | 99.8411288 | 1.144805221 | 7.21E-07 | 0.006476984 |
| MT-TN | 4578.439121 | 3392.950206 | 0.432447566 | 8.94E-07 | 0.006476984 |
| TMSB4XP8 | 326.1623381 | 569.8269881 | -0.806035349 | 3.03E-06 | 0.01474588 |
| RPSAP15 | 679.3093592 | 455.7291849 | 0.576368104 | 3.05E-06 | 0.01474588 |
| RPSAP47 | 189.1091096 | 80.26291476 | 1.237670313 | 6.46E-06 | 0.026743148 |
| MT-TP | 909.0160163 | 648.1317332 | 0.488298623 | 2.03E-05 | 0.073567875 |
| AC116533.1 | 1449.037389 | 1100.131344 | 0.397585653 | 2.93E-05 | 0.09433309 |
| TCEA1P2 | 154.3621475 | 71.27003858 | 1.114143488 | 8.42E-05 | 0.244033232 |
| MT-ND4L | 29762.07201 | 21930.76761 | 0.440534636 | 0.000150039 | 0.395310589 |
| MT-ND3 | 14377.02915 | 11130.72835 | 0.369244703 | 0.000201257 | 0.486068115 |
| MT-TC | 2562.683469 | 2035.198586 | 0.332715283 | 0.000280015 | 0.624262058 |
| NUSAP1 | 279.2225558 | 187.8637479 | 0.572685463 | 0.000377435 | 0.781343863 |
| RPS27 | 6285.413217 | 4863.027086 | 0.370240538 | 0.000613559 | 0.99999426 |
| LINC01669 | 2.700070636 | 18.4049962 | -2.771933309 | 0.000708238 | 0.99999426 |
| KRT86 | 1790.015463 | 2363.246353 | -0.400931751 | 0.00080784 | 0.99999426 |
| CCDC85B | 1159.642052 | 1508.886564 | -0.380164568 | 0.001627825 | 0.99999426 |
| NME2 | 42.37418618 | 78.96440942 | -0.898630928 | 0.001765778 | 0.99999426 |
| MT-ND2 | 38524.42734 | 31797.80888 | 0.276862035 | 0.001997985 | 0.99999426 |
| MIR205HG | 4439.868923 | 3749.753144 | 0.243822611 | 0.002060299 | 0.99999426 |
| MT-CO2 | 138304.5867 | 111307.8503 | 0.313297008 | 0.002085529 | 0.99999426 |
| AC005912.1 | 179.7213995 | 114.3030283 | 0.652989266 | 0.002172828 | 0.99999426 |
| MYH3 | 76.66908973 | 40.6402833 | 0.915180625 | 0.002215159 | 0.99999426 |
| MALAT1 | 7430.721709 | 6262.837822 | 0.246757263 | 0.002299603 | 0.99999426 |
| RGPD5 | 11.05915963 | 32.81688238 | -1.56727863 | 0.002430497 | 0.99999426 |
| MT-ATP6 | 94030.25056 | 74293.02908 | 0.33990307 | 0.003090343 | 0.99999426 |
| TRMT61A | 321.2404908 | 435.243749 | -0.439105333 | 0.003427282 | 0.99999426 |
| MRPL12 | 515.7260564 | 687.4871366 | -0.414793498 | 0.003450942 | 0.99999426 |
| EMP1 | 1682.78234 | 1395.434317 | 0.27037028 | 0.003547217 | 0.99999426 |
| HGH1 | 812.3105538 | 1009.552369 | -0.314073225 | 0.003960172 | 0.99999426 |
| MTND2P28 | 2960.075514 | 1891.326525 | 0.646306687 | 0.004295756 | 0.99999426 |
| AC144530.1 | 105.6236226 | 62.12697298 | 0.766317289 | 0.004403287 | 0.99999426 |
| SDF2L1 | 639.5168856 | 798.151202 | -0.320096575 | 0.004623015 | 0.99999426 |
| MT-RNR1 | 11747.37571 | 9837.861854 | 0.255960133 | 0.004823732 | 0.99999426 |
| RPL18AP3 | 1294.290046 | 1618.745708 | -0.322702242 | 0.004870834 | 0.99999426 |
| FTH1 | 23184.67265 | 19861.96082 | 0.223187483 | 0.005109025 | 0.99999426 |
| RAB5C | 710.104302 | 566.4098364 | 0.32648361 | 0.005663587 | 0.99999426 |
| RGPD8 | 30.42435994 | 11.18187074 | 1.443913296 | 0.005793533 | 0.99999426 |
| RF01972 | 17.79033489 | 39.06314324 | -1.133593825 | 0.005890416 | 0.99999426 |
| MRPL23 | 856.0804515 | 1097.104 | -0.357988916 | 0.005960383 | 0.99999426 |
| MT-ND4 | 201700.8303 | 169052.6162 | 0.254747311 | 0.006006586 | 0.99999426 |
| MAPRE3 | 291.4192995 | 215.6081538 | 0.435513031 | 0.006271751 | 0.99999426 |
| AC048380.2 | 0 | 4.945773501 | -4.735201794 | 0.006458492 | 0.99999426 |
| EEF1A1P5 | 3534.042967 | 2902.186549 | 0.284261774 | 0.006555304 | 0.99999426 |
| OTOF | 0.329768601 | 7.215995976 | -4.319607472 | 0.006911477 | 0.99999426 |
| UBE2V1P2 | 53.55632349 | 28.22559348 | 0.923616527 | 0.007174704 | 0.99999426 |
| MT-CYB | 62836.09538 | 52051.93123 | 0.27164968 | 0.007260731 | 0.99999426 |
| RPL39 | 4208.892261 | 3501.806273 | 0.265464925 | 0.007359864 | 0.99999426 |
| AP001000.1 | 147.090488 | 99.52801478 | 0.563671626 | 0.008007989 | 0.99999426 |
| AL135905.2 | 993.770492 | 793.0645503 | 0.325758074 | 0.008084501 | 0.99999426 |
| AC009318.3 | 0.329768601 | 6.558560982 | -4.181836485 | 0.008257669 | 0.99999426 |
| AC135977.1 | 14.04244583 | 2.305401124 | 2.610514083 | 0.008865729 | 0.99999426 |
| TCIM | 126.7975806 | 74.88354207 | 0.760572406 | 0.009235932 | 0.99999426 |
| MRM1 | 340.7849268 | 429.1522402 | -0.333053769 | 0.009707442 | 0.99999426 |
| ATP5PF | 1156.898631 | 963.0538845 | 0.264717682 | 0.009722825 | 0.99999426 |
| SIVA1 | 1316.324959 | 1583.554273 | -0.266962421 | 0.010002502 | 0.99999426 |
| AC011330.1 | 95.92025469 | 52.14413226 | 0.877077943 | 0.01011491 | 0.99999426 |
| FEM1A | 58.63865574 | 92.76385775 | -0.663194367 | 0.010116126 | 0.99999426 |
| FAM181B | 55.65675102 | 90.0436547 | -0.693429986 | 0.010184089 | 0.99999426 |
| RPL26P19 | 145.7792216 | 97.76748103 | 0.576646032 | 0.010216462 | 0.99999426 |
| INKA2 | 0.332758497 | 6.519286986 | -4.17446555 | 0.010398709 | 0.99999426 |
| AC022540.1 | 4.371547515 | 0 | 4.593689979 | 0.010459339 | 0.99999426 |
| ZNF169 | 129.8069373 | 87.31540135 | 0.573072301 | 0.010498714 | 0.99999426 |
| AC010503.4 | 214.5638327 | 159.7898556 | 0.425723706 | 0.01108625 | 0.99999426 |
| E2F3-IT1 | 4.345923747 | 0 | 4.586548949 | 0.011115719 | 0.99999426 |
| ADRM1 | 1462.51739 | 1688.953761 | -0.207739125 | 0.011185286 | 0.99999426 |
| PWWP2B | 1284.817795 | 1515.831838 | -0.238832909 | 0.011249232 | 0.99999426 |
| AC092384.2 | 20.86586119 | 41.35697441 | -0.987443127 | 0.011622535 | 0.99999426 |
| CENPM | 467.7445461 | 575.6751477 | -0.299208429 | 0.011883683 | 0.99999426 |
| STAG3L1 | 102.4915425 | 51.94030441 | 0.978738542 | 0.012141268 | 0.99999426 |
| FAAP100 | 843.0969796 | 1013.770226 | -0.266300836 | 0.012362296 | 0.99999426 |
| NDUFB1 | 562.9735365 | 448.9913256 | 0.326548357 | 0.012597874 | 0.99999426 |
| MRPL9 | 511.3120661 | 625.1146277 | -0.290220243 | 0.012779926 | 0.99999426 |
| PDF | 245.4426708 | 319.7907755 | -0.382111002 | 0.01332775 | 0.99999426 |
| CEP120 | 452.0578556 | 366.892751 | 0.301342718 | 0.013471167 | 0.99999426 |
| NUDT3 | 204.4892843 | 271.1694287 | -0.407221124 | 0.013529386 | 0.99999426 |
| KRT87P | 1089.465516 | 687.3351804 | 0.664323177 | 0.013540466 | 0.99999426 |
| MACC1 | 739.9054502 | 620.738054 | 0.253238515 | 0.013578777 | 0.99999426 |
| STUB1 | 894.496899 | 1083.214975 | -0.276427024 | 0.013911922 | 0.99999426 |
| ADCK2 | 1101.390563 | 1274.491474 | -0.21061288 | 0.013921135 | 0.99999426 |
| Z84485.1 | 28.37880888 | 11.44414081 | 1.310412762 | 0.014391251 | 0.99999426 |
| NT5C3AP1 | 29.86227283 | 11.1337158 | 1.4229613 | 0.014791046 | 0.99999426 |
| MT-RNR2 | 94554.57337 | 79617.36238 | 0.248071586 | 0.01480397 | 0.99999426 |
| RPL29 | 11837.32863 | 10261.74657 | 0.206121854 | 0.014908899 | 0.99999426 |
| FGFR4 | 22.8337985 | 44.23156769 | -0.953728574 | 0.015505069 | 0.99999426 |
| IMP3 | 620.9600509 | 755.248073 | -0.283082135 | 0.0159944 | 0.99999426 |
| AC246785.2 | 0.665516994 | 7.236915654 | -3.431734367 | 0.016182362 | 0.99999426 |
| MAZ | 1351.559013 | 1587.660674 | -0.232627061 | 0.016199966 | 0.99999426 |
| UBE2CP2 | 12.12405145 | 2.633242892 | 2.204854894 | 0.016725829 | 0.99999426 |
| AMDHD2 | 194.9550005 | 256.5304511 | -0.396322428 | 0.017025028 | 0.99999426 |
| DGKQ | 654.4554825 | 778.9197932 | -0.251023227 | 0.017341344 | 0.99999426 |
| FXYD5 | 1898.586692 | 1636.666462 | 0.214354279 | 0.017427669 | 0.99999426 |
| PEMT | 321.7515228 | 399.9232629 | -0.314401052 | 0.017445188 | 0.99999426 |
| AL732292.2 | 5.368537713 | 0.334502476 | 3.928818285 | 0.017661449 | 0.99999426 |
| SLC25A10 | 295.1033424 | 371.6934505 | -0.333061533 | 0.017744304 | 0.99999426 |
| USP27X-AS1 | 1.008949781 | 7.533081689 | -2.90127811 | 0.018062888 | 0.99999426 |
| SERHL | 18.24906096 | 5.59607901 | 1.705968094 | 0.018452275 | 0.99999426 |
| RPL23A | 5138.675808 | 4458.933926 | 0.204777489 | 0.018609479 | 0.99999426 |
| GATD3A | 826.1212155 | 1043.661541 | -0.336652587 | 0.018622844 | 0.99999426 |
| THAP11 | 603.7363668 | 724.7724774 | -0.263910564 | 0.018673474 | 0.99999426 |
| SMG1P2 | 88.45514533 | 55.7256149 | 0.664984486 | 0.018920966 | 0.99999426 |
| THORLNC | 6.708541388 | 0.649836731 | 3.358795948 | 0.019074434 | 0.99999426 |
| ZNF706 | 811.0665427 | 689.9120286 | 0.233243946 | 0.019379594 | 0.99999426 |
| ADAP1 | 623.0715994 | 745.036768 | -0.258441251 | 0.019693026 | 0.99999426 |
| SIRT7 | 490.2276539 | 594.9638397 | -0.27996462 | 0.020082591 | 0.99999426 |
| FNDC8 | 0 | 3.903460856 | -4.396695825 | 0.020195943 | 0.99999426 |
| VWA1 | 1582.441909 | 1818.135383 | -0.200206857 | 0.020206254 | 0.99999426 |
| TMPRSS7 | 0.332758497 | 5.220082302 | -3.853798571 | 0.020576528 | 0.99999426 |
| TMPOP2 | 0.659537203 | 6.859167489 | -3.360534944 | 0.020818028 | 0.99999426 |
| MT1X | 307.4859004 | 407.3862255 | -0.40651773 | 0.020876622 | 0.99999426 |
| ZSCAN26 | 183.3349429 | 133.4022827 | 0.459083878 | 0.020990965 | 0.99999426 |
| TPGS1 | 166.7015403 | 221.5701728 | -0.409910323 | 0.021008304 | 0.99999426 |
| CALM2P2 | 28.82686066 | 13.44846665 | 1.100936034 | 0.021126086 | 0.99999426 |
| PPP1R16A | 493.5807593 | 598.5693663 | -0.278387677 | 0.021351935 | 0.99999426 |
| ALDH1A1 | 29.26295285 | 51.84973443 | -0.825439184 | 0.021364487 | 0.99999426 |
| GRIK4 | 5.055423193 | 0.32737299 | 3.840974352 | 0.021667427 | 0.99999426 |
| ENDOG | 324.358934 | 402.7503915 | -0.31287253 | 0.021708011 | 0.99999426 |
| PTPRM | 495.0434449 | 400.9486611 | 0.304636474 | 0.022121671 | 0.99999426 |
| AP000560.1 | 11.48885272 | 2.940978884 | 1.961395182 | 0.022401649 | 0.99999426 |
| MAPK10 | 84.31067176 | 51.05730799 | 0.723959091 | 0.022686926 | 0.99999426 |
| AL513314.2 | 3.023859446 | 11.75631727 | -1.959903835 | 0.023781769 | 0.99999426 |
| ACAP3 | 330.1554602 | 405.7467785 | -0.298091008 | 0.02385591 | 0.99999426 |
| RPL32 | 9435.793823 | 8274.609705 | 0.189512352 | 0.02394562 | 0.99999426 |
| MT-ND1 | 20602.80265 | 17796.40979 | 0.211274784 | 0.024008556 | 0.99999426 |
| MT-CO3 | 135293.1037 | 114612.076 | 0.239332118 | 0.024044216 | 0.99999426 |
| AC018638.1 | 79.80920462 | 115.6101764 | -0.534088227 | 0.024347931 | 0.99999426 |
| ZNF37A | 328.7814333 | 263.4220013 | 0.319752216 | 0.024872043 | 0.99999426 |
| AC012085.1 | 90.70896498 | 143.7965217 | -0.664813797 | 0.025014679 | 0.99999426 |
| RPS10P3 | 53.25143622 | 31.18130011 | 0.773704243 | 0.025281861 | 0.99999426 |
| TFAP2E | 4.079781575 | 13.74907315 | -1.760953161 | 0.025526893 | 0.99999426 |
| ITGA10 | 9.743075124 | 23.34586046 | -1.259670157 | 0.025661362 | 0.99999426 |
| RPL38 | 3313.683058 | 2803.01982 | 0.241516617 | 0.025799508 | 0.99999426 |
| COMMD6 | 294.7065109 | 394.7609584 | -0.421709847 | 0.026021763 | 0.99999426 |
| MT-ND5 | 61215.23956 | 53220.30111 | 0.201921487 | 0.026049604 | 0.99999426 |
| RNA5SP97 | 3.98114238 | 0 | 4.461208297 | 0.026415944 | 0.99999426 |
| PARP10 | 1175.466318 | 1346.323328 | -0.195623729 | 0.026423184 | 0.99999426 |
| AC244154.1 | 105.7920877 | 70.8568812 | 0.579463917 | 0.026568816 | 0.99999426 |
| GJC2 | 7.746524143 | 1.318841682 | 2.558035491 | 0.026719708 | 0.99999426 |
| EIF4BP6 | 69.09518237 | 43.66062408 | 0.663372305 | 0.02678532 | 0.99999426 |
| AC106772.1 | 0 | 3.624242806 | -4.286882885 | 0.02680347 | 0.99999426 |
| AC008083.1 | 0 | 3.612204071 | -4.282791172 | 0.026807623 | 0.99999426 |
| NR2F6 | 3059.725989 | 3456.542471 | -0.175932847 | 0.027148824 | 0.99999426 |
| FP565260.3 | 17.52120283 | 34.55484281 | -0.979985696 | 0.02715014 | 0.99999426 |
| AC116407.1 | 24.71975071 | 10.49369757 | 1.237817713 | 0.02724452 | 0.99999426 |
| EIF3C | 490.3709336 | 241.131332 | 1.024193332 | 0.027315384 | 0.99999426 |
| ZNF480 | 426.8608966 | 349.6280051 | 0.288012468 | 0.027530813 | 0.99999426 |
| SAPCD2 | 1535.026378 | 1806.576024 | -0.235263721 | 0.027601714 | 0.99999426 |
| JAG2 | 4942.550873 | 5635.219193 | -0.189287687 | 0.027779695 | 0.99999426 |
| FZD5 | 289.9829387 | 362.0914255 | -0.320660799 | 0.028115332 | 0.99999426 |
| BX255925.3 | 419.0276928 | 502.4609694 | -0.262338068 | 0.028234904 | 0.99999426 |
| RPUSD1 | 773.3378339 | 930.0623337 | -0.266766408 | 0.028242055 | 0.99999426 |
| SEC61G | 1092.350754 | 932.8925543 | 0.227794463 | 0.028526225 | 0.99999426 |
| SERF1B | 22.11320546 | 142.1890027 | -2.684744916 | 0.028579303 | 0.99999426 |
| DMPK | 1443.617515 | 1635.480179 | -0.18003502 | 0.028660837 | 0.99999426 |
| KDM7A | 250.4999222 | 192.9344561 | 0.377099561 | 0.028744795 | 0.99999426 |
| DALRD3 | 544.514873 | 646.2832941 | -0.247209215 | 0.02909674 | 0.99999426 |
| DCXR | 1084.167175 | 1241.230317 | -0.195185194 | 0.029293457 | 0.99999426 |
| TRIM28 | 12336.28492 | 13828.29497 | -0.164753997 | 0.029334052 | 0.99999426 |
| UFSP1 | 45.89776425 | 71.47901389 | -0.641143369 | 0.029413894 | 0.99999426 |
| FBXL6 | 669.13998 | 783.7942795 | -0.228339983 | 0.030075014 | 0.99999426 |
| MAMDC2-AS1 | 3.043503423 | 11.20547943 | -1.883291554 | 0.030080033 | 0.99999426 |
| RPL37 | 13235.60839 | 11424.35373 | 0.212340277 | 0.030207534 | 0.99999426 |
| AC011405.1 | 10.08949781 | 2.007014853 | 2.343005685 | 0.030246939 | 0.99999426 |
| AC004471.1 | 3.345943653 | 0 | 4.209128318 | 0.030642275 | 0.99999426 |
| HNRNPM | 6553.282803 | 7512.906807 | -0.197236923 | 0.030800835 | 0.99999426 |
| PPARG | 218.9186026 | 170.5489811 | 0.360008759 | 0.030891736 | 0.99999426 |
| IFITM1 | 3397.796896 | 2818.42936 | 0.26989158 | 0.031385793 | 0.99999426 |
| NUDCD3 | 2109.818191 | 2376.891355 | -0.172108835 | 0.031940615 | 0.99999426 |
| Z99496.1 | 0.662527098 | 5.908255473 | -3.142000238 | 0.031975454 | 0.99999426 |
| AC006511.5 | 3.348933549 | 0 | 4.210192848 | 0.032015021 | 0.99999426 |
| RFXANK | 574.667826 | 671.4242913 | -0.224618968 | 0.032047928 | 0.99999426 |
| ZNF821 | 61.1388774 | 37.40629617 | 0.709142049 | 0.032139903 | 0.99999426 |
| IER2 | 1784.422271 | 1572.758513 | 0.182288056 | 0.032236934 | 0.99999426 |
| RF01974 | 3.728664394 | 0 | 4.362751785 | 0.032536966 | 0.99999426 |
| SCRIB | 3778.806639 | 4264.353586 | -0.17448649 | 0.032592565 | 0.99999426 |
| DZANK1 | 12.79255834 | 27.2572647 | -1.091759217 | 0.032867923 | 0.99999426 |
| AP001178.1 | 12.06341492 | 3.600634114 | 1.743813481 | 0.032933768 | 0.99999426 |
| RPL10P9 | 2062.673817 | 1710.418433 | 0.27028714 | 0.032958476 | 0.99999426 |
| KLHL2P1 | 0.998275491 | 7.205239921 | -2.843208071 | 0.033766866 | 0.99999426 |
| NOTCH1 | 2486.254061 | 2872.934862 | -0.20866042 | 0.033818572 | 0.99999426 |
| GNLY | 414.0231594 | 323.5174299 | 0.356757646 | 0.033882841 | 0.99999426 |
| AL451064.2 | 9.809691449 | 2.293831167 | 2.093911009 | 0.033883797 | 0.99999426 |
| CHPF | 6566.584719 | 7352.13736 | -0.163020455 | 0.034005568 | 0.99999426 |
| AL031590.1 | 0.346422682 | 4.577843835 | -3.663835193 | 0.034167817 | 0.99999426 |
| WDR34 | 2387.889162 | 2700.356994 | -0.177448834 | 0.034190611 | 0.99999426 |
| AC093904.3 | 0 | 3.27057211 | -4.140366072 | 0.034606708 | 0.99999426 |
| AC099518.4 | 4.398875886 | 0.322463741 | 3.639224975 | 0.035091768 | 0.99999426 |
| AC138866.1 | 2.394640511 | 10.22873849 | -2.105448467 | 0.03516202 | 0.99999426 |
| PRELID1 | 1379.33723 | 1629.382551 | -0.240487665 | 0.035286369 | 0.99999426 |
| HEATR1 | 1090.646346 | 1239.927388 | -0.185092816 | 0.035571025 | 0.99999426 |
| FAM173A | 439.7903438 | 527.6824539 | -0.262900437 | 0.035789418 | 0.99999426 |
| LINC01277 | 3.651373779 | 0 | 4.336371696 | 0.03592533 | 0.99999426 |
| OBSCN-AS1 | 24.12811512 | 10.4637733 | 1.204596547 | 0.036102747 | 0.99999426 |
| RPL13P12 | 693.059565 | 872.4563698 | -0.332125995 | 0.036609041 | 0.99999426 |
| TRAF4 | 2956.43059 | 3298.840999 | -0.158232724 | 0.036878699 | 0.99999426 |
| TWF1P1 | 74.20301485 | 48.86106938 | 0.601461815 | 0.036911024 | 0.99999426 |
| ERI3 | 903.9953801 | 1039.795388 | -0.202185643 | 0.036987423 | 0.99999426 |
| STAT2 | 1432.820881 | 1246.31712 | 0.201498368 | 0.038193671 | 0.99999426 |
| AC116913.1 | 26.16308808 | 12.76298249 | 1.034988601 | 0.038573789 | 0.99999426 |
| ALDH3A2 | 2591.967675 | 2885.378195 | -0.154791003 | 0.038781383 | 0.99999426 |
| SNORA66 | 3.750012974 | 12.12962497 | -1.703123906 | 0.039325987 | 0.99999426 |
| ARHGAP31-AS1 | 0.329768601 | 4.264729815 | -3.560806604 | 0.039402686 | 0.99999426 |
| FP565260.1 | 167.3170677 | 118.8258092 | 0.493696372 | 0.039516353 | 0.99999426 |
| TMEM238 | 1611.868186 | 1874.139574 | -0.217499143 | 0.039682147 | 0.99999426 |
| RPL7P26 | 8.387702661 | 1.980717147 | 2.08773906 | 0.039877867 | 0.99999426 |
| HMGB1P6 | 1004.040141 | 1184.059002 | -0.238336665 | 0.04047908 | 0.99999426 |
| EIF4A1P10 | 147.4245318 | 110.7607127 | 0.411638884 | 0.040587483 | 0.99999426 |
| SLC4A1APP1 | 4.00249096 | 12.445428 | -1.631902215 | 0.040835183 | 0.99999426 |
| TACO1 | 553.8152097 | 644.3595059 | -0.218649757 | 0.041005252 | 0.99999426 |
| ASAP1 | 1083.374295 | 935.3472149 | 0.21194137 | 0.041085587 | 0.99999426 |
| FTH1P8 | 103.6530185 | 67.64228376 | 0.615099436 | 0.041197508 | 0.99999426 |
| AC026801.2 | 35.8997641 | 19.74206855 | 0.864933922 | 0.041220608 | 0.99999426 |
| NPDC1 | 1671.181535 | 1868.551619 | -0.161092357 | 0.041409052 | 0.99999426 |
| ANXA2P2 | 673.0238172 | 790.4249416 | -0.232557884 | 0.04148433 | 0.99999426 |
| HIF1AP1 | 3.026849342 | 0 | 4.063319731 | 0.041630538 | 0.99999426 |
| SLC27A4 | 854.6382143 | 997.5250151 | -0.223379515 | 0.041755394 | 0.99999426 |
| TTC9C | 636.6516758 | 536.1329303 | 0.248440677 | 0.041764047 | 0.99999426 |
| RPL23 | 6973.785373 | 6221.121847 | 0.164796429 | 0.041881616 | 0.99999426 |
| ZP1 | 0.679181179 | 5.562183041 | -3.044117085 | 0.041884469 | 0.99999426 |
| PCGF2 | 628.4570797 | 535.7846544 | 0.230451611 | 0.042011109 | 0.99999426 |
| CCL28 | 170.0706333 | 120.4207446 | 0.499670674 | 0.042041294 | 0.99999426 |
| DGCR6L | 206.7043387 | 259.0208737 | -0.32619818 | 0.042081219 | 0.99999426 |
| PRIM1 | 185.6761157 | 235.9163637 | -0.346717817 | 0.042172596 | 0.99999426 |
| RNA5SP203 | 4.327565063 | 0.322463741 | 3.619762445 | 0.042292829 | 0.99999426 |
| AL158801.2 | 4.31560548 | 0.334502476 | 3.616429046 | 0.042436281 | 0.99999426 |
| AC131392.1 | 16.67921317 | 5.57200154 | 1.578412569 | 0.04278148 | 0.99999426 |
| Z68871.1 | 36.60328375 | 20.62296839 | 0.826992735 | 0.042915694 | 0.99999426 |
| IQCA1 | 130.427929 | 97.17352116 | 0.424963783 | 0.043010481 | 0.99999426 |
| SPINK8 | 3.013185156 | 0 | 4.057869518 | 0.04302676 | 0.99999426 |
| CREB3 | 826.7159243 | 949.3479181 | -0.199517626 | 0.043164629 | 0.99999426 |
| DOHH | 380.5437014 | 470.8620546 | -0.308252905 | 0.043390689 | 0.99999426 |
| PCCA | 489.3379696 | 408.0407501 | 0.26248941 | 0.04351214 | 0.99999426 |
| MIR3157 | 3.409570083 | 0 | 4.232582154 | 0.043569718 | 0.99999426 |
| CD320 | 861.5581031 | 1033.188155 | -0.262185548 | 0.0437114 | 0.99999426 |
| REPIN1 | 4603.13767 | 5080.604773 | -0.142460463 | 0.043719486 | 0.99999426 |
| CLP1 | 411.565154 | 487.3133551 | -0.24397807 | 0.044029333 | 0.99999426 |
| CACNA1A | 3.329289572 | 11.42497259 | -1.773905296 | 0.044438662 | 0.99999426 |
| AL596244.1 | 24.69316478 | 11.19168924 | 1.141592638 | 0.044506761 | 0.99999426 |
| U47924.1 | 1.022613966 | 6.512626278 | -2.684583859 | 0.044515772 | 0.99999426 |
| RNU6-638P | 3.007205365 | 0 | 4.055431379 | 0.044689038 | 0.99999426 |
| C19orf24 | 698.4976888 | 818.4923779 | -0.229052897 | 0.044925674 | 0.99999426 |
| POLRMT | 1664.489607 | 1874.513366 | -0.171603441 | 0.044964198 | 0.99999426 |
| ZNF696 | 173.0207421 | 220.456318 | -0.350480273 | 0.044988518 | 0.99999426 |
| SLC30A1 | 1216.725665 | 1381.356907 | -0.183287095 | 0.045025757 | 0.99999426 |
| AC013643.3 | 3.054177713 | 0 | 4.074267811 | 0.045079589 | 0.99999426 |
| C5orf38 | 1810.300493 | 2024.017003 | -0.1609177 | 0.045400302 | 0.99999426 |
| IL1B | 434.2299575 | 334.589909 | 0.37633851 | 0.045451131 | 0.99999426 |
| AC137932.2 | 4.055443099 | 0.32737299 | 3.522293634 | 0.046434544 | 0.99999426 |
| AGBL5 | 600.9715354 | 518.3083511 | 0.213534262 | 0.046522755 | 0.99999426 |
| RBBP4P5 | 2.996531075 | 0 | 4.05110057 | 0.046692132 | 0.99999426 |
| TMEM102 | 394.8891231 | 467.1049505 | -0.241936092 | 0.046771315 | 0.99999426 |
| PPIAP22 | 251.5146047 | 196.9003141 | 0.352362783 | 0.046818259 | 0.99999426 |
| TPM1-AS | 2.048217828 | 8.551316864 | -2.072383826 | 0.046955018 | 0.99999426 |
| AC106869.1 | 0.346422682 | 4.654516716 | -3.684414399 | 0.046980257 | 0.99999426 |
| TNC | 184.0713787 | 141.4849028 | 0.378696844 | 0.047251364 | 0.99999426 |
| AC016907.2 | 4.029819331 | 0.334502476 | 3.514791878 | 0.047381011 | 0.99999426 |
| TATDN2 | 510.7996526 | 603.2454028 | -0.240431712 | 0.047495326 | 0.99999426 |
| EDDM13 | 34.35084558 | 18.35765517 | 0.903093847 | 0.047508201 | 0.99999426 |
| ALDH16A1 | 994.861517 | 1141.996143 | -0.199072048 | 0.047759907 | 0.99999426 |
| RGS2 | 135.6525597 | 101.6024597 | 0.417010791 | 0.047795188 | 0.99999426 |
| COX8A | 1986.906722 | 2261.896447 | -0.187026339 | 0.047862182 | 0.99999426 |
| RPSAP58 | 461.8350775 | 569.1795946 | -0.301742496 | 0.04802794 | 0.99999426 |
| AC025031.2 | 0.329768601 | 4.543947866 | -3.654437384 | 0.048099315 | 0.99999426 |
| AC017081.1 | 3.3066557 | 0 | 4.194049681 | 0.048121132 | 0.99999426 |
| AC006026.3 | 0 | 2.940978884 | -3.987334178 | 0.048307403 | 0.99999426 |
| MCAT | 340.7920957 | 410.3580495 | -0.268766986 | 0.048326966 | 0.99999426 |
| SUMO2P19 | 10.01818698 | 2.61582613 | 1.936963209 | 0.048388319 | 0.99999426 |
| SEMA3B | 14451.96881 | 15796.39373 | -0.128332982 | 0.048652616 | 0.99999426 |
| TELO2 | 1166.909711 | 1342.189558 | -0.202085583 | 0.049171605 | 0.99999426 |
| ADAMTS7P3 | 2.990551284 | 0 | 4.048586725 | 0.049230928 | 0.99999426 |
| CR382285.1 | 3.330994175 | 0 | 4.20319618 | 0.049373981 | 0.99999426 |
| IRF2BP1 | 847.1467315 | 968.8156611 | -0.193804775 | 0.049472613 | 0.99999426 |
| LRRC45 | 1008.893599 | 1174.739368 | -0.220022392 | 0.049523622 | 0.99999426 |
| VARS | 3154.577111 | 3581.524404 | -0.183279736 | 0.049789727 | 0.99999426 |
| AC009283.1 | 17.86164572 | 7.5856771 | 1.237819767 | 0.049831352 | 0.99999426 |
| GTF2H2B | 55.98555745 | 82.92993906 | -0.568422406 | 0.049838878 | 0.99999426 |
| PPP1R35 | 778.828792 | 911.7539373 | -0.227312567 | 0.049947814 | 0.99999426 |
