# Supplementary figures and images for: Extracellular vesicles isolated from hyperuricemia patients might aggravate airway inflammation of COPD via senescence-associated pathway
Source: J Inflamm (Lond). 2022 Nov 2;19:18. doi: 10.1186/s12950-022-00315-w (PMC9628085; doi:10.1186/s12950-022-00315-w)

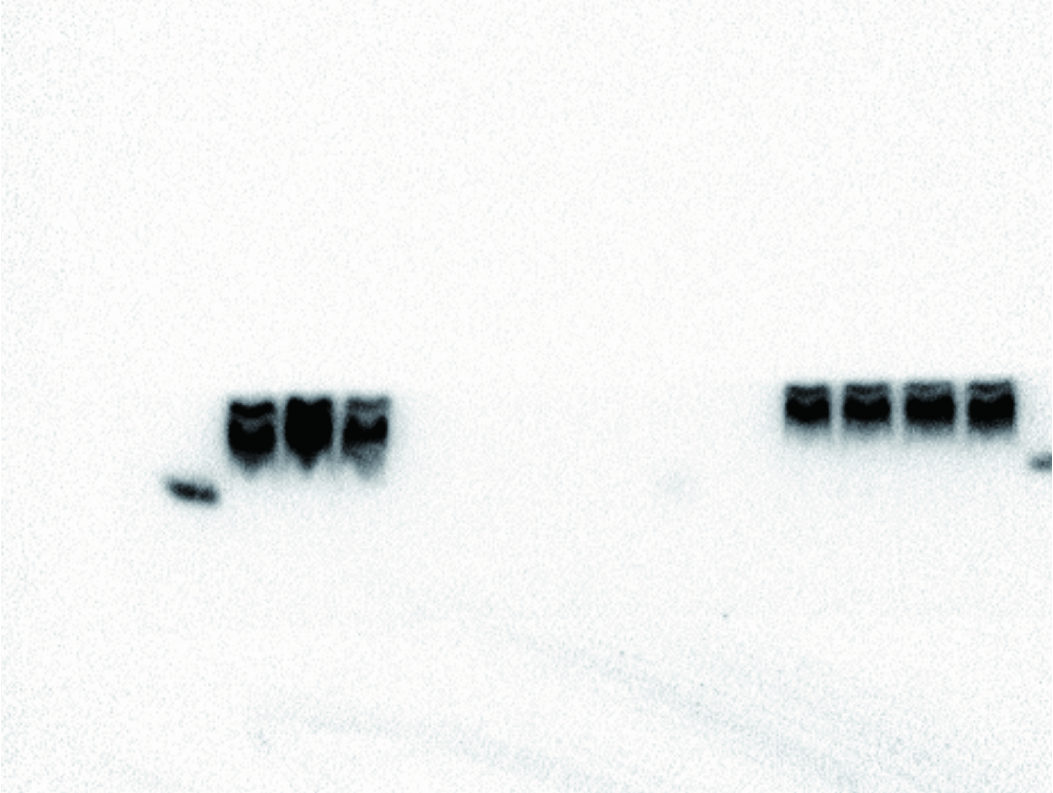

Supplement: Supplementary file 2 — Additional file 2. [file 12950_2022_315_MOESM2_ESM.zip › westernblot_original/Figure4_Sputum_IL8.tif]

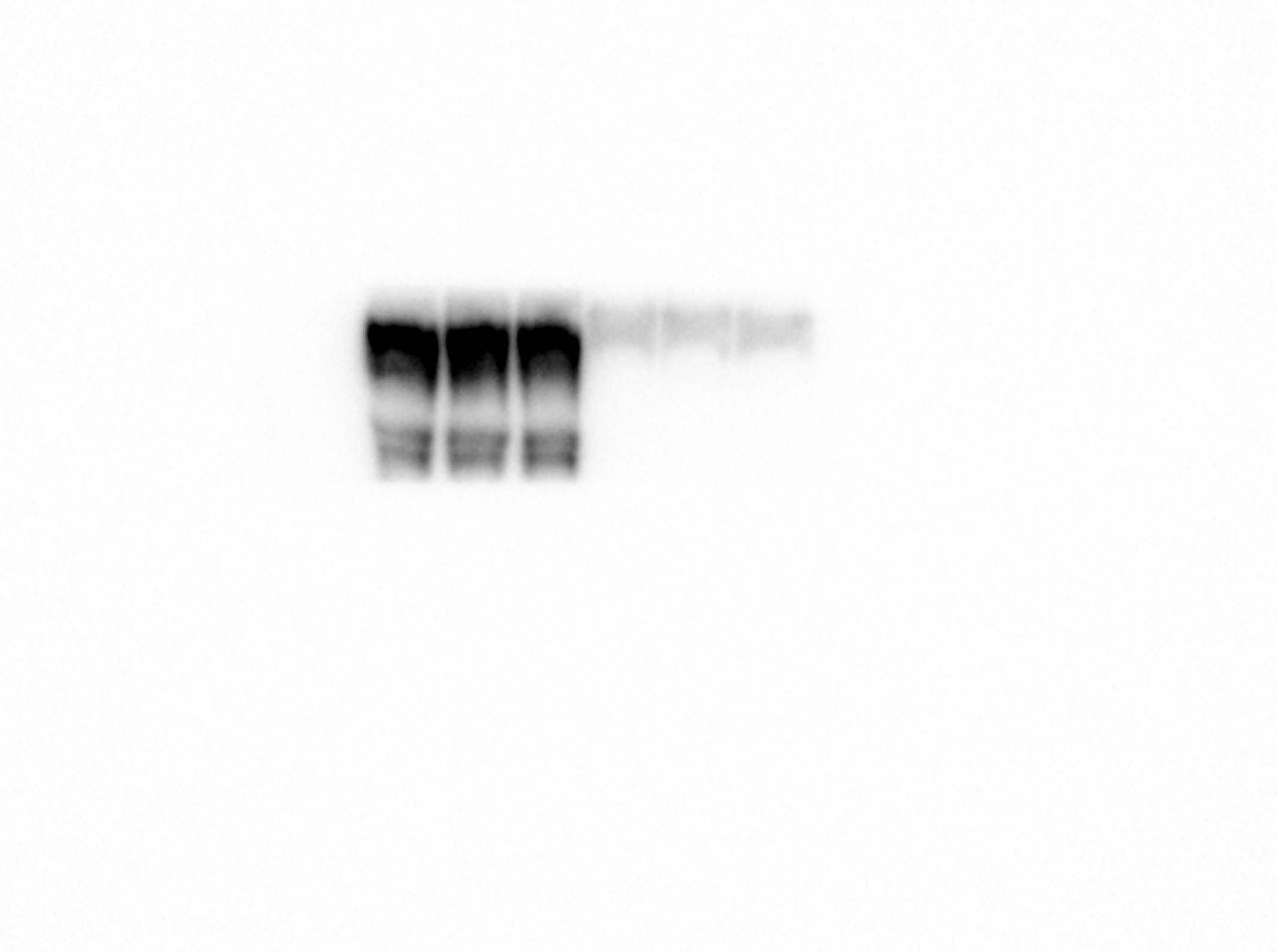

Supplement: Supplementary file 2 — Additional file 2. [file 12950_2022_315_MOESM2_ESM.zip › westernblot_original/Figure4_Sputum_Anti.tif]

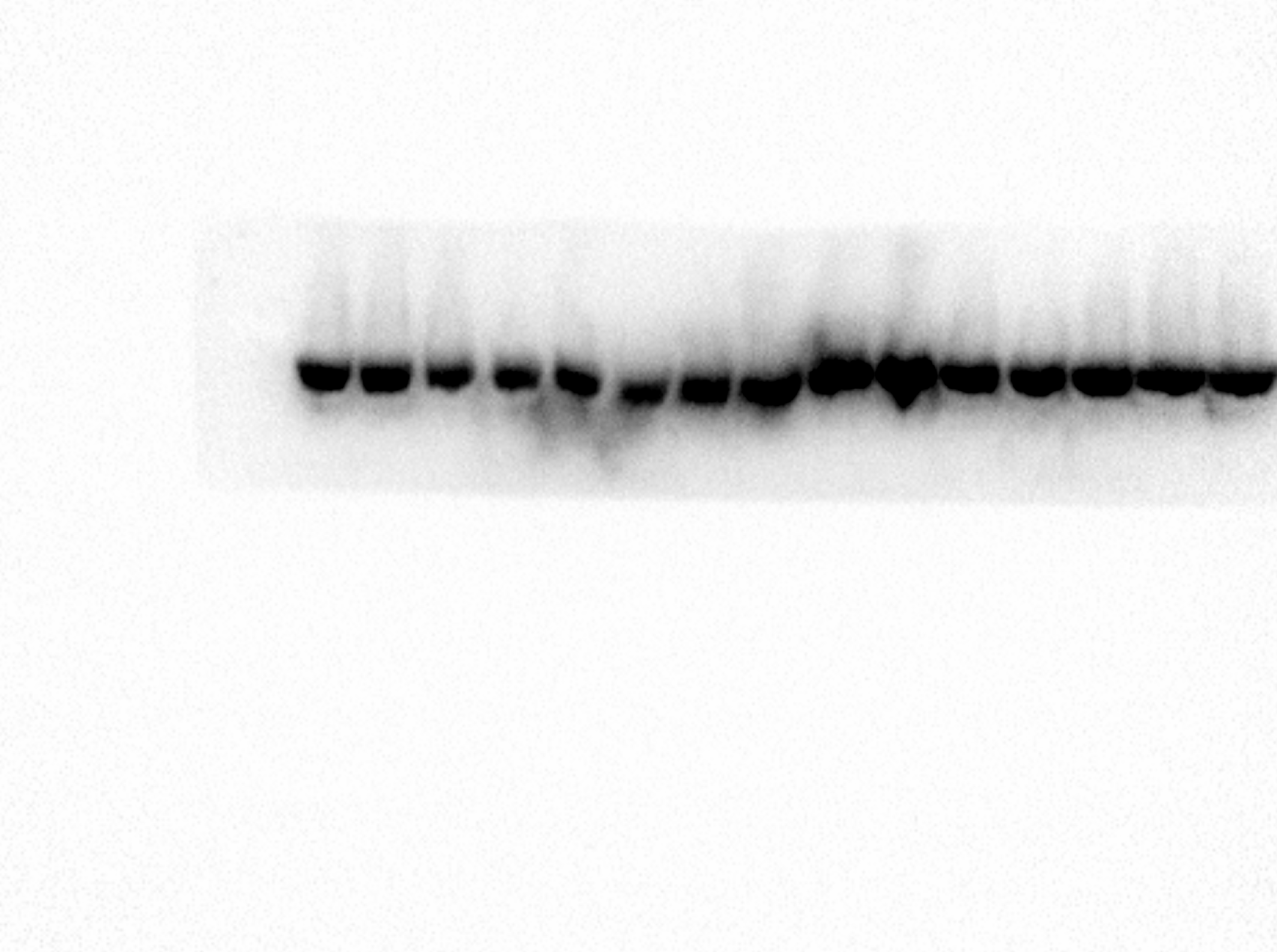

Supplement: Supplementary file 2 — Additional file 2. [file 12950_2022_315_MOESM2_ESM.zip › westernblot_original/Figure4_Serum_IL6.tif]

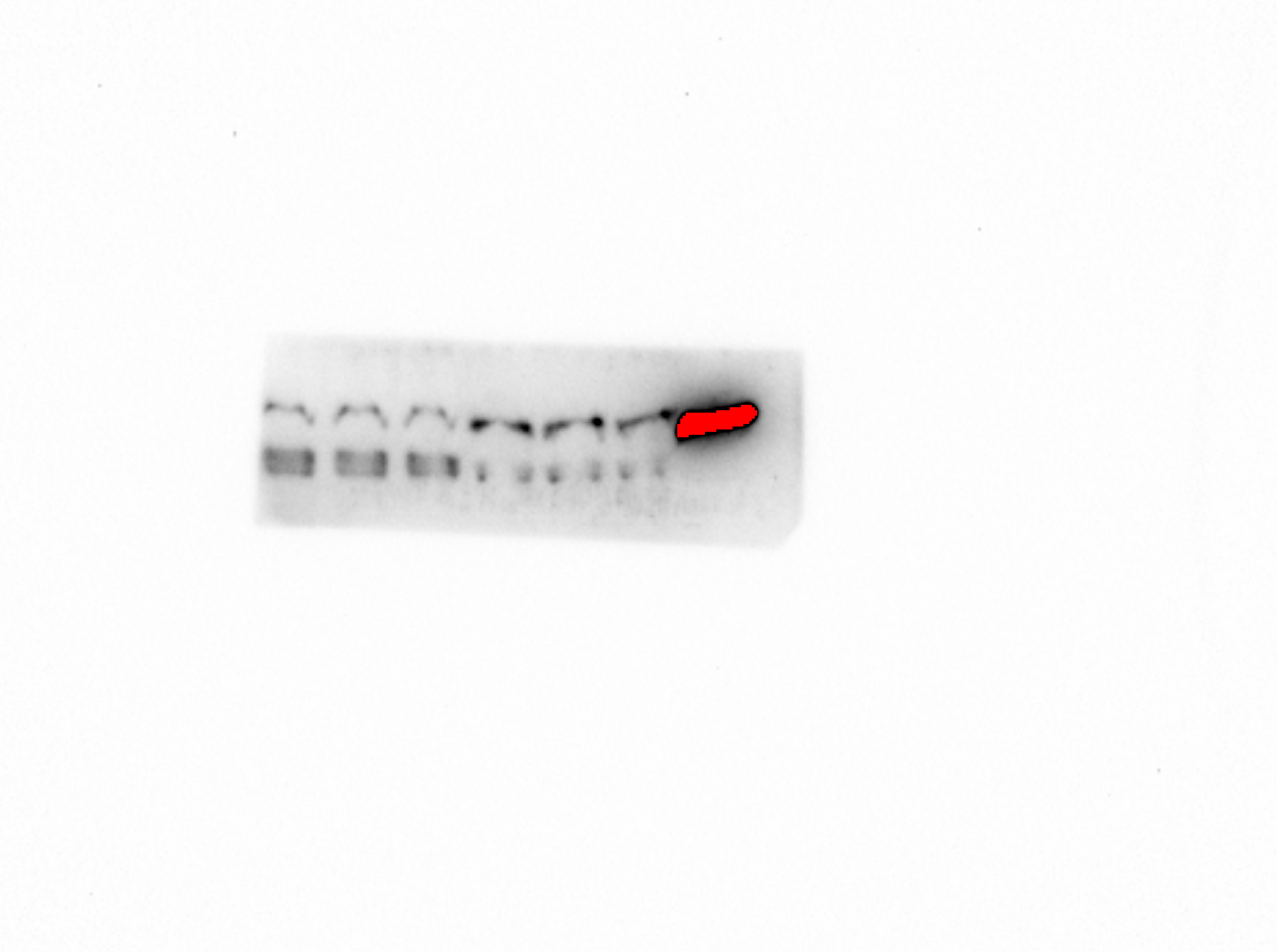

Supplement: Supplementary file 2 — Additional file 2. [file 12950_2022_315_MOESM2_ESM.zip › westernblot_original/Figure4_Sputum_MUC1.tif]

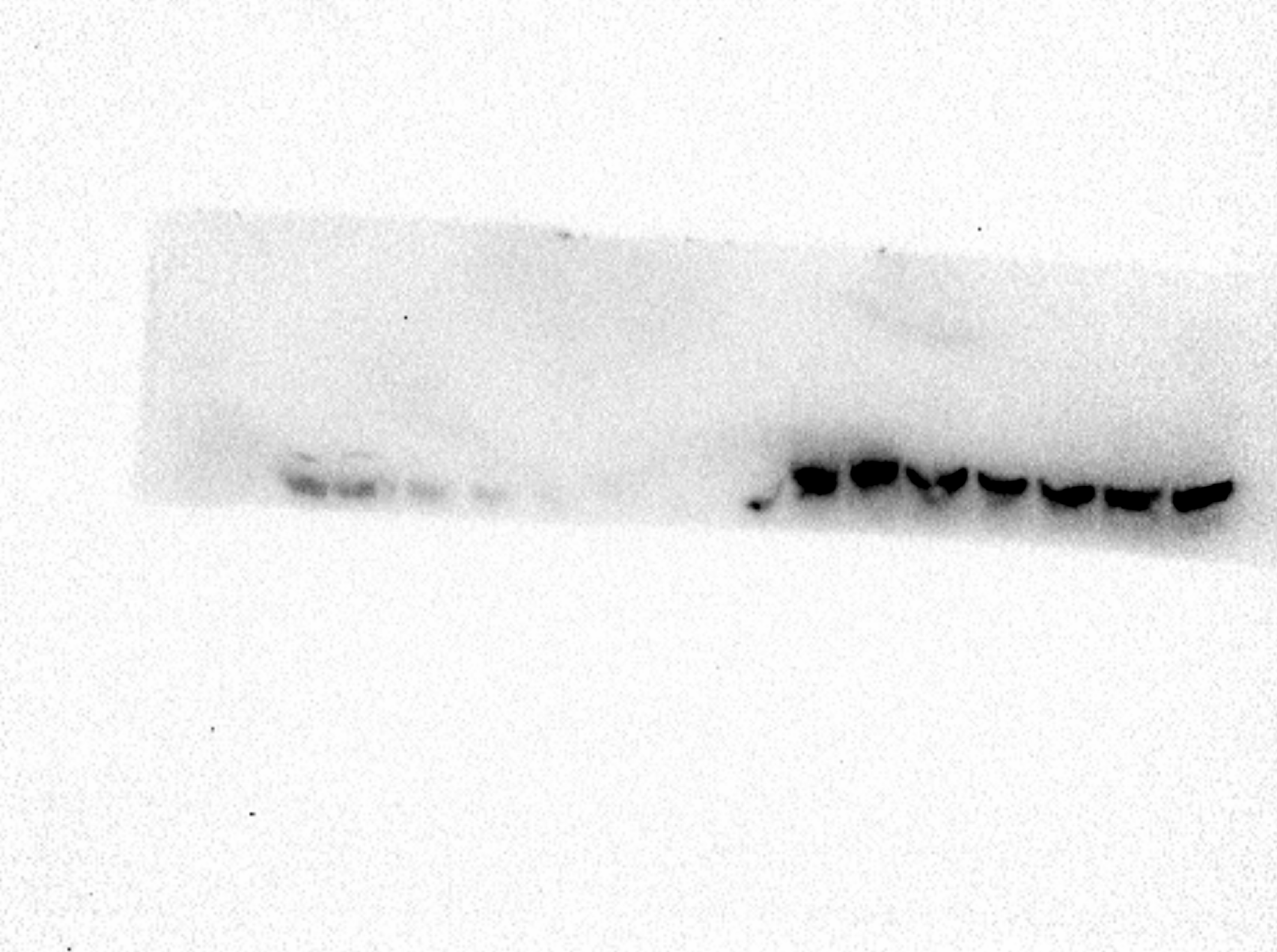

Supplement: Supplementary file 2 — Additional file 2. [file 12950_2022_315_MOESM2_ESM.zip › westernblot_original/Figure4_Serum_ALNE.tif]

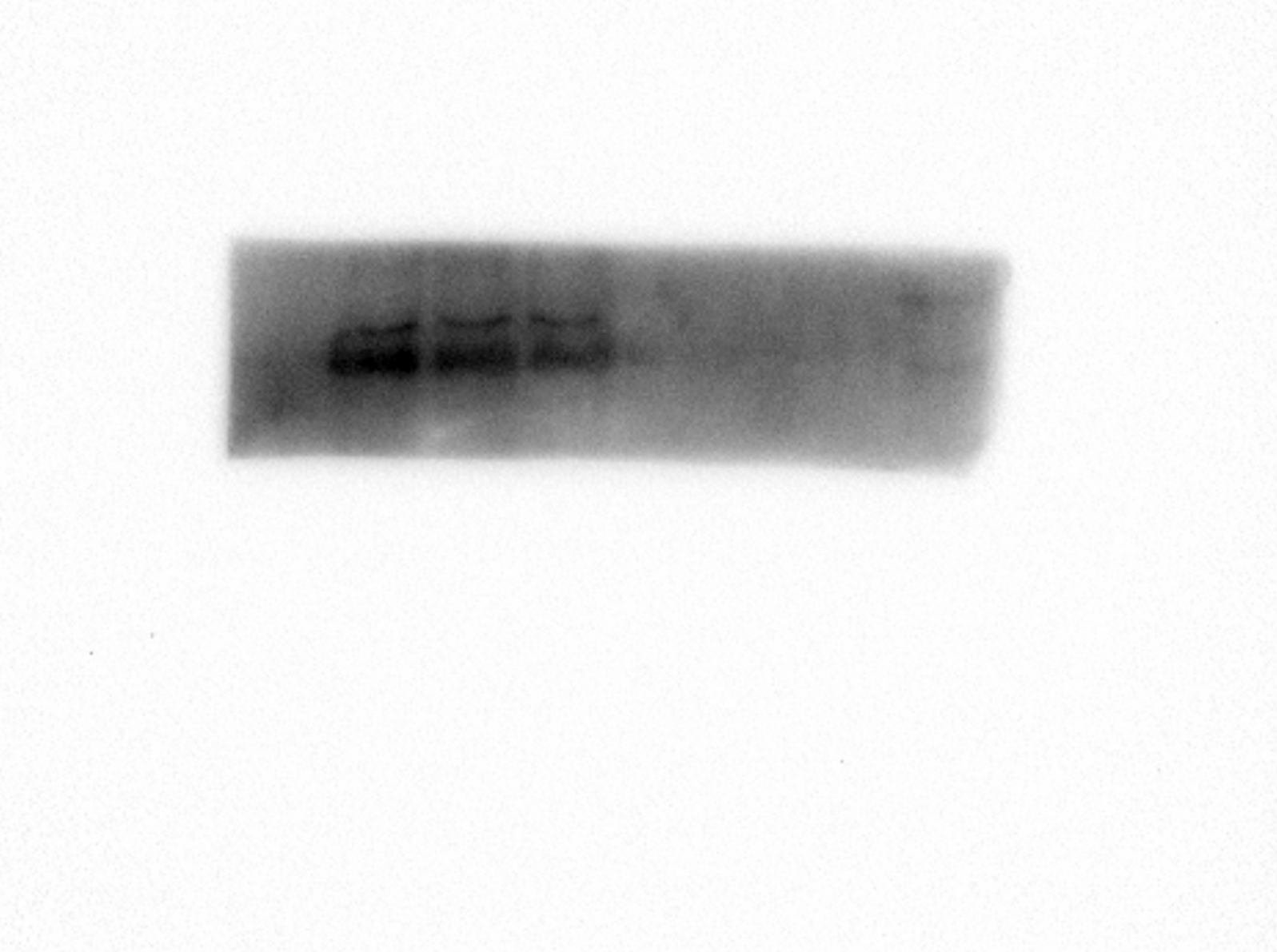

Supplement: Supplementary file 2 — Additional file 2. [file 12950_2022_315_MOESM2_ESM.zip › westernblot_original/Figure4_BALF_IL6.tif]

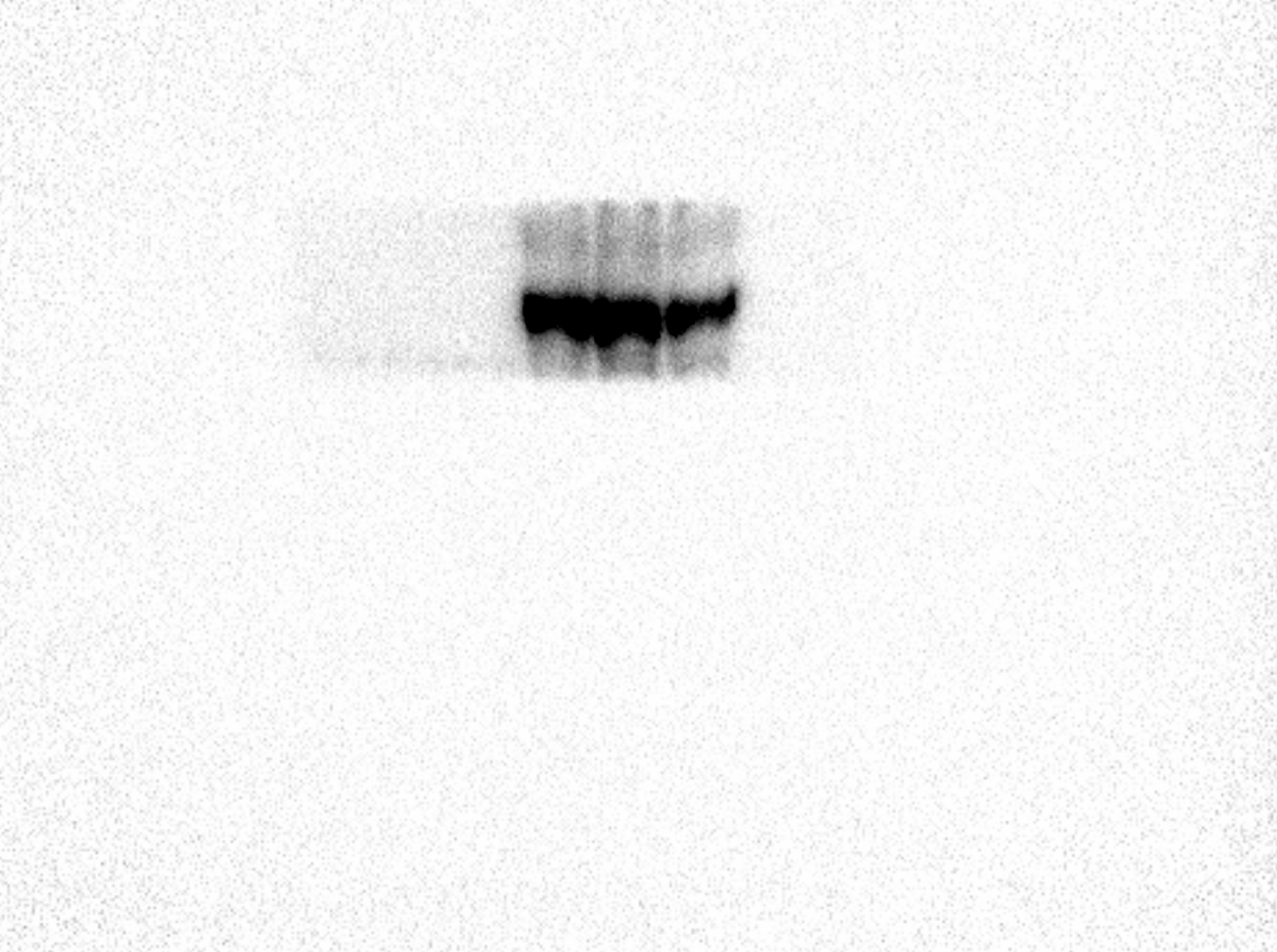

Supplement: Supplementary file 2 — Additional file 2. [file 12950_2022_315_MOESM2_ESM.zip › westernblot_original/Figure4_BALF_Anti.tif]

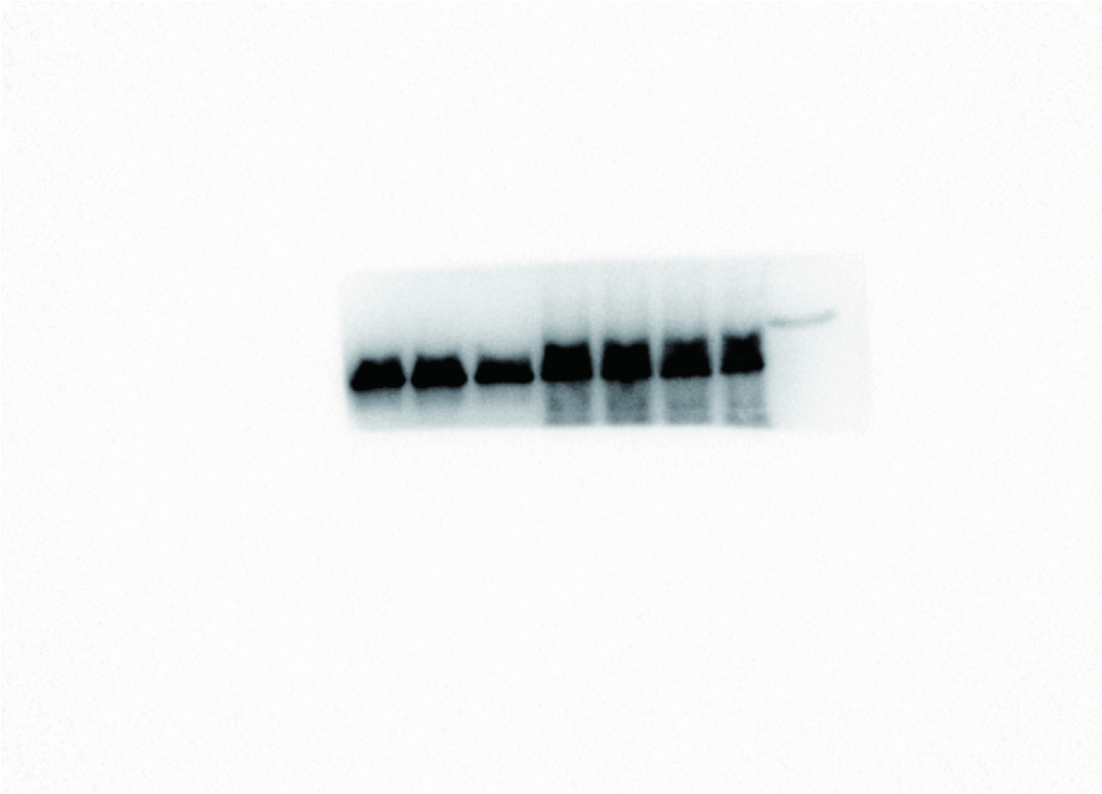

Supplement: Supplementary file 2 — Additional file 2. [file 12950_2022_315_MOESM2_ESM.zip › westernblot_original/Figure4_BALF_ALNE.tif]

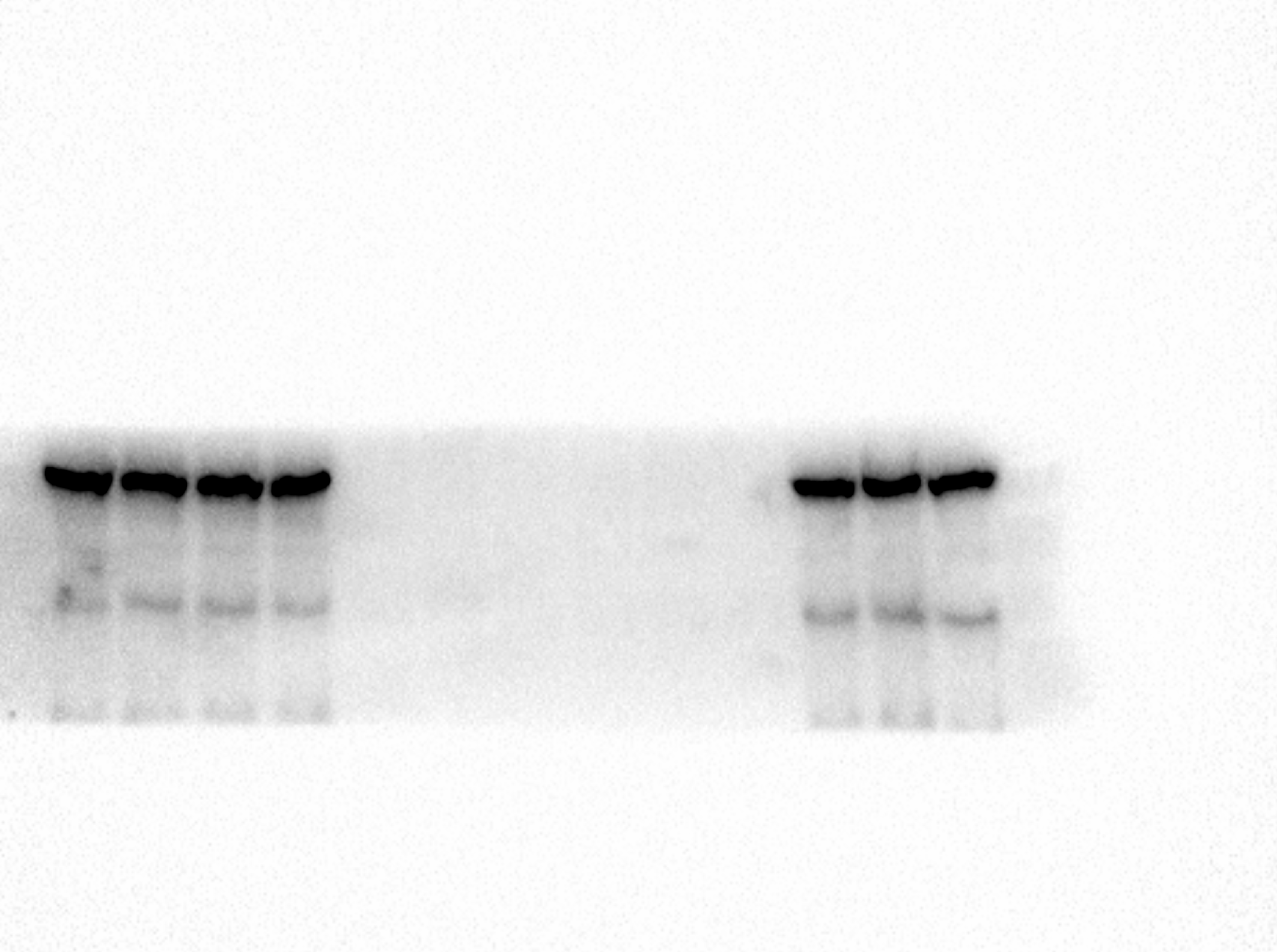

Supplement: Supplementary file 2 — Additional file 2. [file 12950_2022_315_MOESM2_ESM.zip › westernblot_original/Figure4_Sputum_IL6.tif]

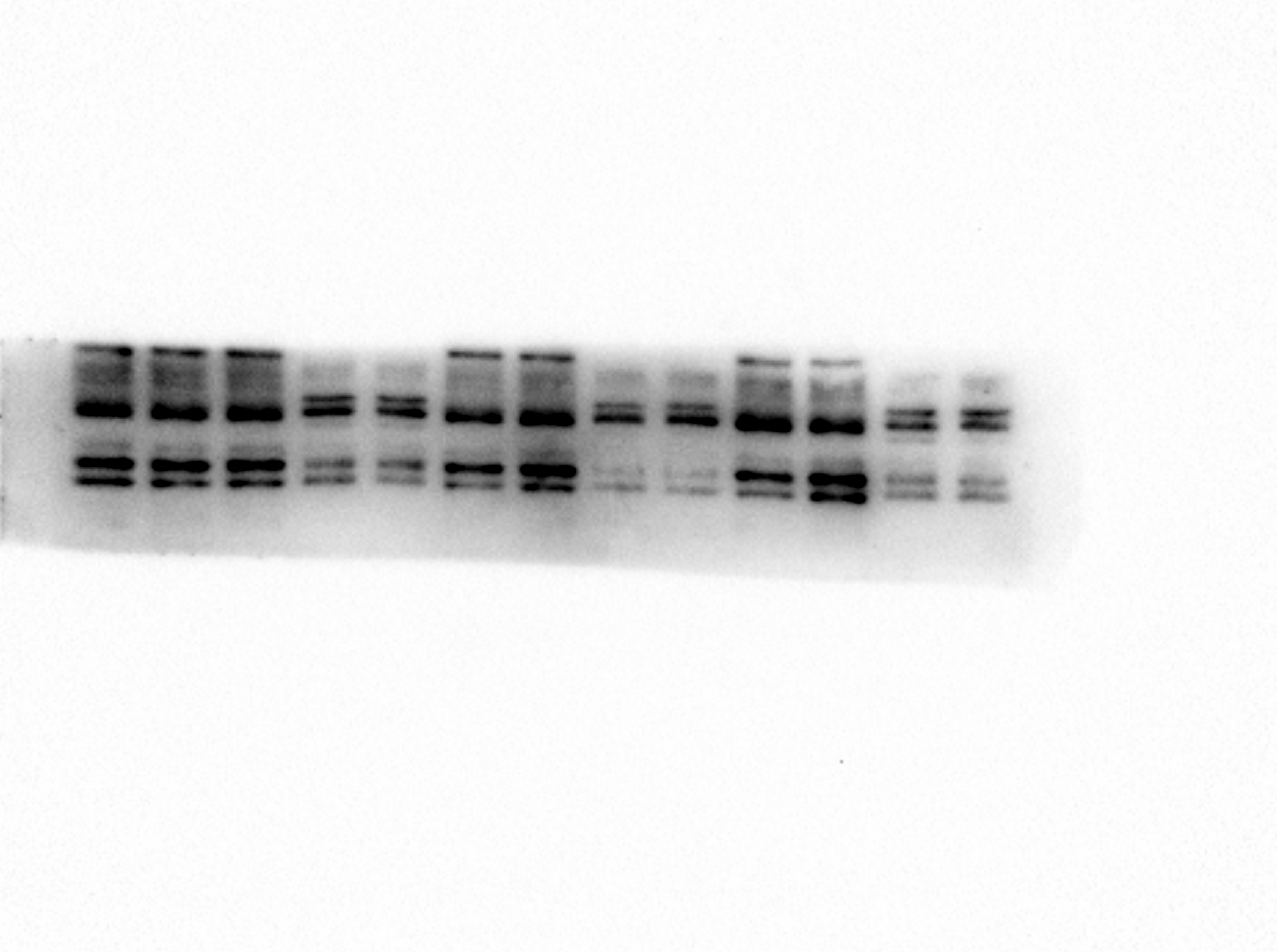

Supplement: Supplementary file 2 — Additional file 2. [file 12950_2022_315_MOESM2_ESM.zip › westernblot_original/Figure5_IL1a.tif]

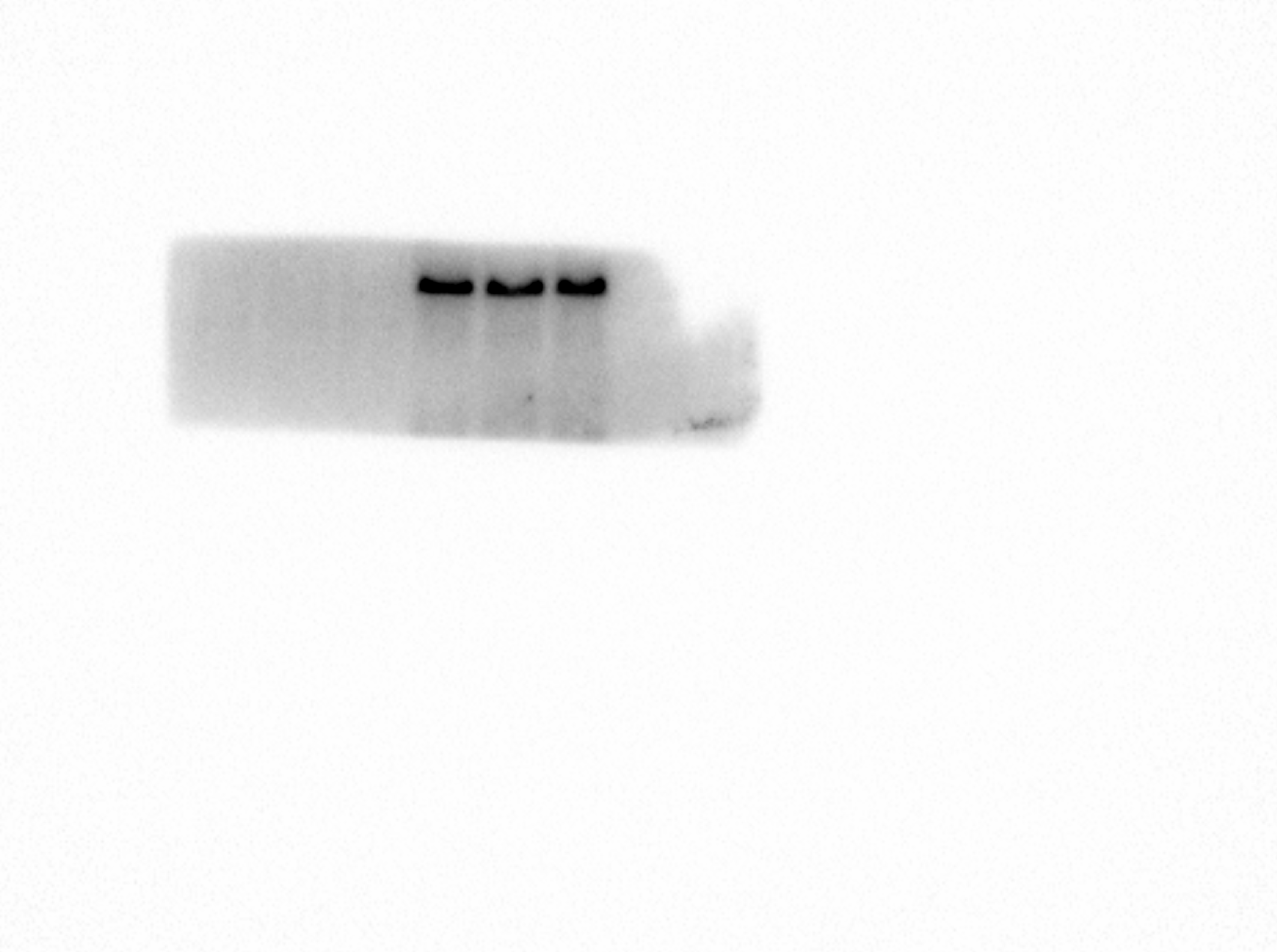

Supplement: Supplementary file 2 — Additional file 2. [file 12950_2022_315_MOESM2_ESM.zip › westernblot_original/Figure4_BALF_MUC1.tif]

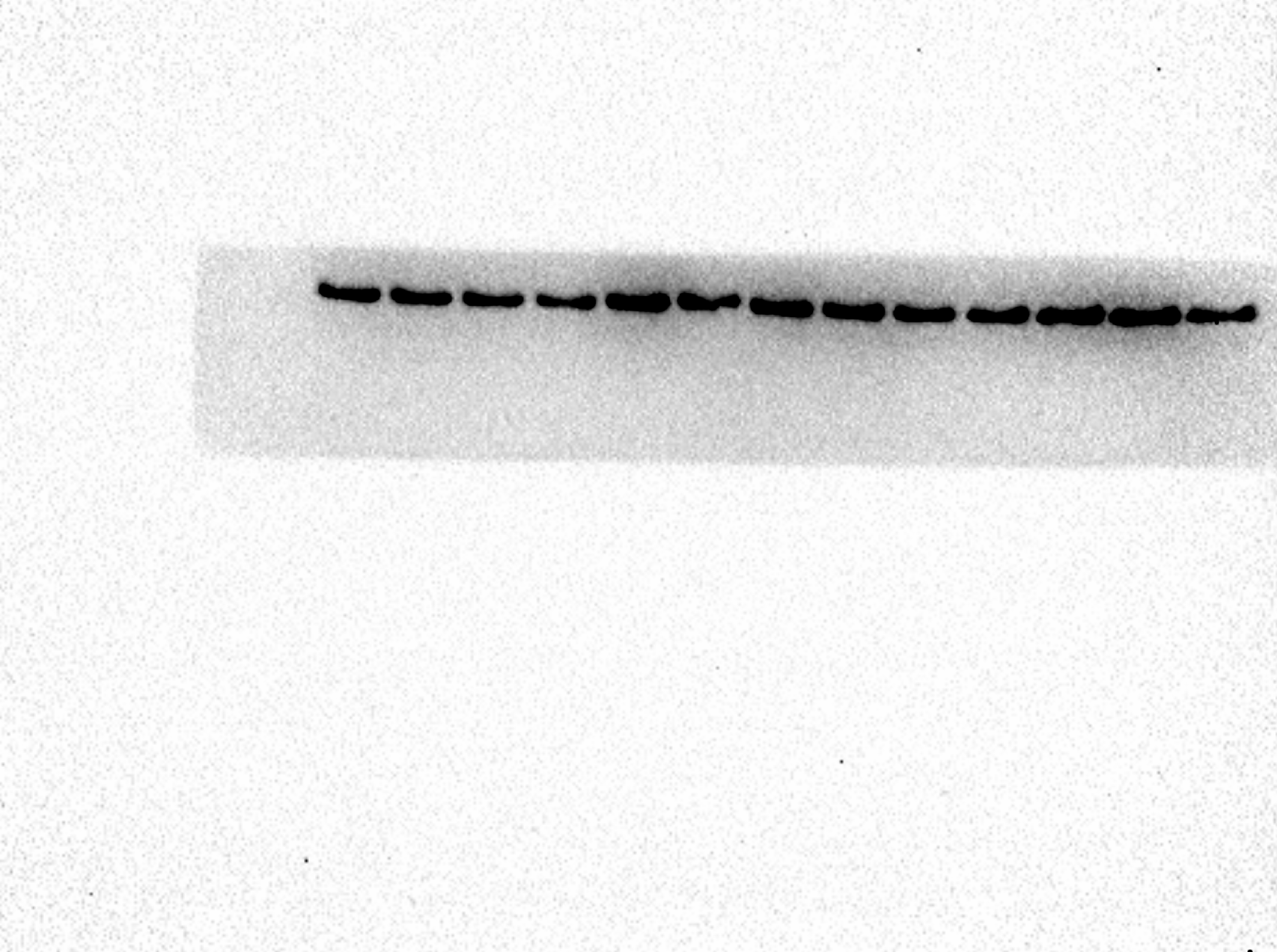

Supplement: Supplementary file 2 — Additional file 2. [file 12950_2022_315_MOESM2_ESM.zip › westernblot_original/Figure4_Serum_GD.tif]

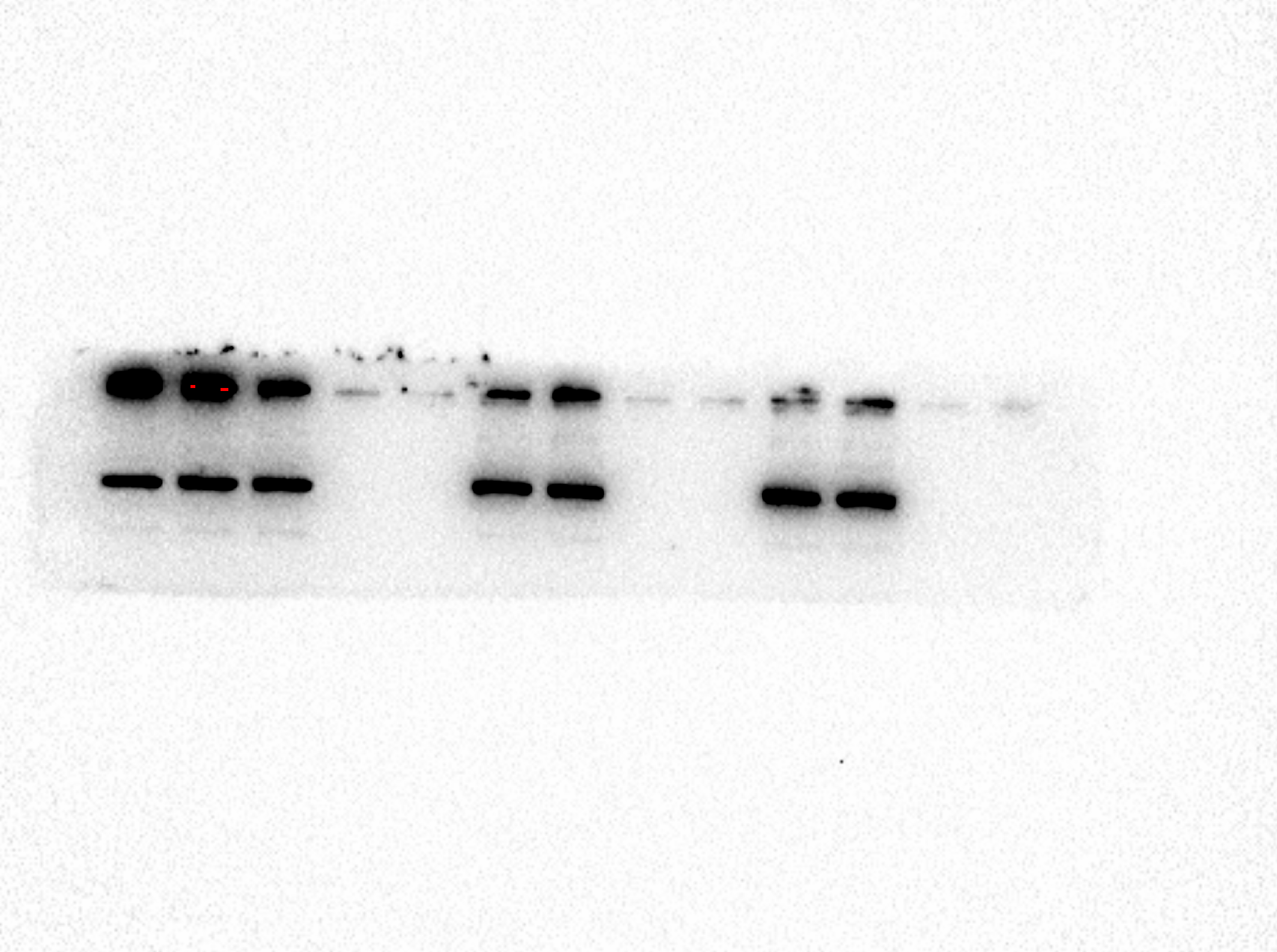

Supplement: Supplementary file 2 — Additional file 2. [file 12950_2022_315_MOESM2_ESM.zip › westernblot_original/Figure5_53BP1.tif]

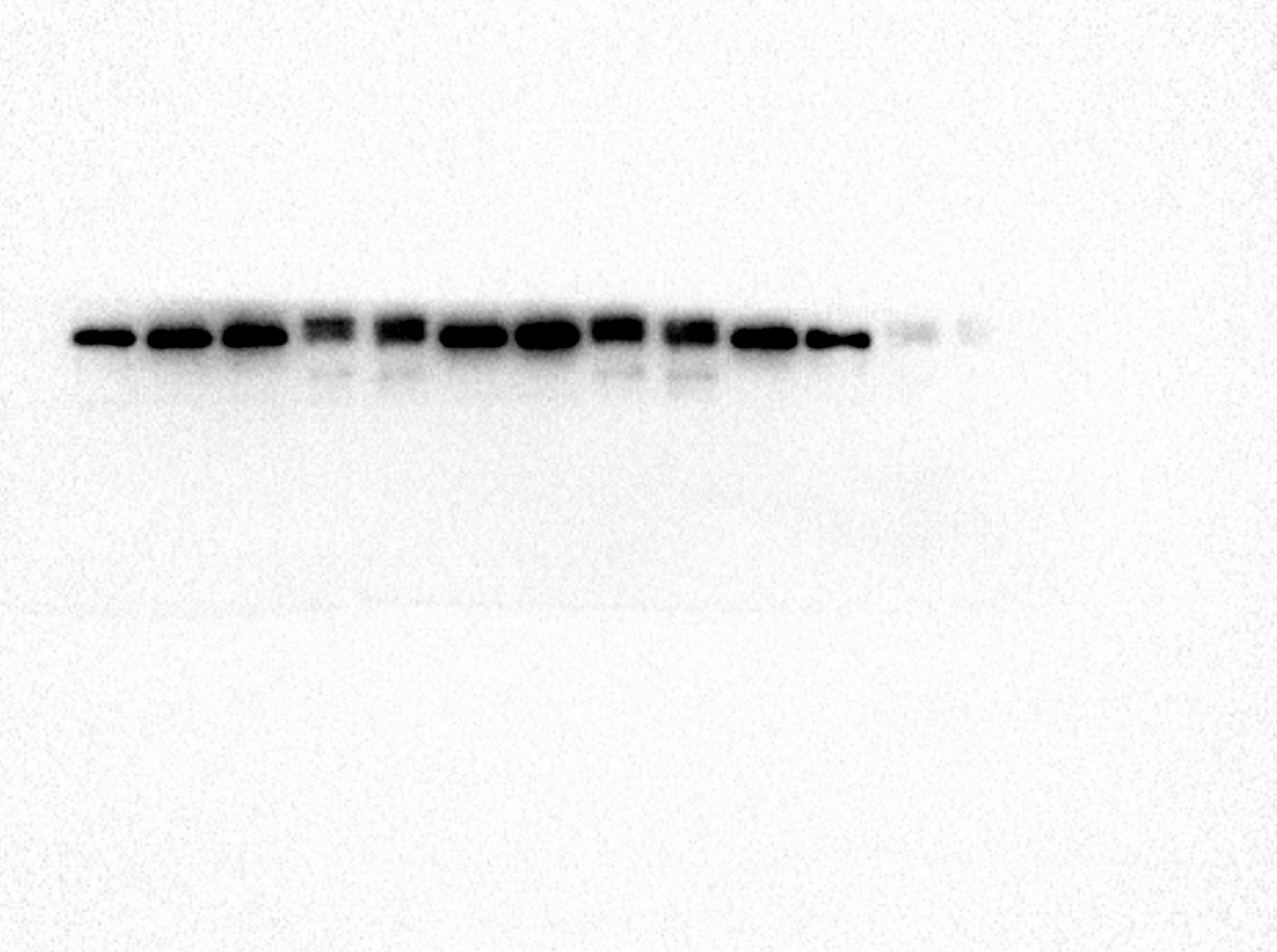

Supplement: Supplementary file 2 — Additional file 2. [file 12950_2022_315_MOESM2_ESM.zip › westernblot_original/Figure5_p21CIP1.tif]
